# The Ni(II)-Binding Activity of the Intrinsically Disordered Region of Human NDRG1, a Protein Involved in Cancer Development

**DOI:** 10.3390/biom12091272

**Published:** 2022-09-09

**Authors:** Ylenia Beniamino, Vittoria Cenni, Mario Piccioli, Stefano Ciurli, Barbara Zambelli

**Affiliations:** 1 Laboratory of Bioinorganic Chemistry, Department of Pharmacy and Biotechnology, University of Bologna, Viale Giuseppe Fanin 40, 40127 Bologna, Italy; 2 CNR Institute of Molecular Genetics “Luigi-Luca Cavalli-Sforza” Unit of Bologna, Via di Barbiano 1/10, 40136 Bologna, Italy; 3 Department of Chemistry, Center for Magnetic Resonance, University of Florence, 50121 Florence, Italy

**Keywords:** nickel, intrinsically disordered regions, lung cancer, nmr, isothermal titration calorimetry, circular dichroism, light scattering

## Abstract

Nickel exposure is associated with tumors of the respiratory tract such as lung and nasal cancers, acting through still-uncharacterized mechanisms. Understanding the molecular basis of nickel-induced carcinogenesis requires unraveling the mode and the effects of Ni(II) binding to its intracellular targets. A possible Ni(II)-binding protein and a potential focus for cancer treatment is *h*NDRG1, a protein induced by Ni(II) through the hypoxia response pathway, whose expression correlates with higher cancer aggressiveness and resistance to chemotherapy in lung tissue. The protein sequence contains a unique C-terminal sequence of 83 residues (*h*NDRG1*C), featuring a three-times-repeated decapeptide, involved in metal binding, lipid interaction and post-translational phosphorylation. In the present work, the biochemical and biophysical characterization of unmodified *h*NDRG1*C was performed. Bioinformatic analysis assigned it to the family of the intrinsically disordered regions and the absence of secondary and tertiary structure was experimentally proven by circular dichroism and NMR. Isothermal titration calorimetry revealed the occurrence of a Ni(II)-binding event with micromolar affinity. Detailed information on the Ni(II)-binding site and on the residues involved was obtained in an extensive NMR study, revealing an octahedral paramagnetic metal coordination that does not cause any major change of the protein backbone, which is coherent with CD analysis. *h*NDRG1*C was found in a monomeric form by light-scattering experiments, while the full-length *h*NDRG1 monomer was found in equilibrium between the dimer and tetramer, both in solution and in human cell lines. The results are the first essential step for understanding the cellular function of *h*NDRG1*C at the molecular level, with potential future applications to clarify its role and the role of Ni(II) in cancer development.

## 1. Introduction

Lung cancer is the first cause of tumor-related death worldwide [1]. The major risk factor is cigarette smoke, but exposition to air pollution also significantly increases tumor incidence: in particular, according to the World Health Organization (WHO), inhalation of fine dusts is responsible for more than three million deaths every year, mostly due to cancer [2]. One of the dangerous elements present both in cigarette smoke and in fine dusts is nickel. In 1990, compounds containing this metal were classified as class I carcinogens by WHO [3]. Moreover, occupational exposure to nickel compounds in nickel mines or refineries increases nasal and lung cancer [4]. Understanding the mechanisms of nickel-induced carcinogenesis is therefore of social importance, especially considering the ever-growing level of air pollution [5], and would allow for new drugs to be found to prevent or to cure lung cancer.

Exposure to nickel activates the cell hypoxia response, a tumorigenic state that allows the transformed cells to grow faster due to increased blood supply, promoting the transcription of several genes from the hypoxia-recognizing elements (HREs) [6]: Ni(II) ions likely substitute Fe(II) in the non-heme metal center of asparaginyl and prolyl hydroxylases [6]. These enzymes hydroxylate the regulatory subunit of the hypoxia-inducing factor 1 (HIF-1) [7], named HIF-1α, driving its ubiquitination and rapid proteasomal degradation [8]. Their inhibition, caused by metal substitution, stabilizes HIF-1α, which translocates into the nucleus, where it interacts with HIF-1β and promotes HIF-dependent transcription from HREs [6]. Imitation of the state of hypoxia by nickel compounds is likely involved in the nickel-induced carcinogenic transformation, as it may select cells with altered energy metabolism, changed growth control and/or resistance to apoptosis.

Among Ni(II)- and hypoxia-induced genes, the one known as human N-myc downstream-regulated gene 1 (*h*NDRG1) is particularly noteworthy [9]. Its transcription is repressed by the C-Myc and N-myc oncogenes, often upregulated in cancers [10]. As Myc overexpression is linked to cell proliferation and metastasis, the genes repressed by Myc, including that coding for *h*NDRG1, are believed to regulate tumor progression [11]. The *h*NDRG1 protein is predominantly cytosolic [12], but it translocates into the nucleus in response to DNA damage in different cell lines, and it was also found in mitochondria and membranes, suggesting a function in stress response and DNA repair [13,14]. A large bulk of literature proposes for *h*NDRG1 a multiplicity of functions, including embryogenesis and development, cell growth and differentiation, lipid biosynthesis and myelination, stress responses, vesicle sorting and trafficking, and immunity [15], rendering it a central regulator of cellular biochemistry.

*h*NDRG1 is a key regulator of multiple signaling pathways that modulate tumor progression [14]. Interestingly, the protein shows a strictly tissue-specific pleiotropic activity in carcinogenesis: it works as metastasis suppressor in brain [16], breast [17], colon [18], glioma [19] and prostate cancers [14], as it suppresses the TGF-β/SMAD pathway by reducing the expression of the downstream targets SMAD2 and SMAD3 [20], as well as the Wnt/β-catenin pathway by interacting with the Wnt co-receptor LRP6 [21]. On the other hand, its overexpression in cervical [22], hepatocellular [23], renal [24] and lung [25] cancers is associated with poor prognosis and higher tumor aggressiveness [26], making this protein a negative prognostic biomarker for these tumors. The molecular role of *h*NDRG1 in worsening the tumor outcomes in these tissues is not known.

In the non-small-cell lung carcinoma (NSCLC), higher expression of *h*NDRG1 correlates with higher cancer aggressiveness and resistance to chemotherapy, especially with cisplatin medication [25]. It also favors stem-like properties in tumor-initiating cells (TICs) by interacting with the Skp2 kinase and preventing the degradation of C-Myc through Skp2-mediated ubiquitination [27]. For these reasons, *h*NDRG1 has been proposed as a possible target for both treating tumor aggressiveness and modulating the cellular effects of nickel compounds in lung cells [28]. Interestingly, *h*NDRG1 showed Ni(II)-binding properties [29], suggesting a possible link between the oncologic potential of nickel for lung cells and the tumor response. A function for this protein as a modulator of nickel-dependent toxicity, through Ni(II) chelation and detoxification, has been proposed [30].

Despite the large number of studies that revealed the multiplicity of cellular pathways regulated by *h*NDRG1, its mechanism of action in the cellular machinery remains elusive. Understanding the precise function that this protein plays in the carcinogenic process at the cellular, molecular and structural level is necessary to design potential drugs that modulate or inhibit its specific role in tumors.

*h*NDRG1 belongs to the *h*NDRG family, which contains four orthologues, namely *h*NDRG1, *h*NDRG2, *h*NDRG3 and *h*NDRG4. All these proteins are encoded by genes repressed by Myc and share approximately 60% sequence identity, with a nonenzymatic α/β hydrolase-fold usually linked to protein scaffold or regulation of protein–protein interactions [31,32]. The crystallographic structures of *h*NDRG2, *h*NDRG3 and more recently *h*NDRG1, all artificially truncated at the N- and C-termini to favor crystallization, were determined [33,34,35]. They all contain a common α/β hydrolase fold made of six β-strands surrounded by eight α-helices.

Like other *h*NDRG proteins, *h*NDRG1 contains a N-terminal flexible region of 32 residues, not present in the crystal structures due to the truncation, which is quite conserved between *h*NDRG1 and *h*NDRG3. This sequence contains several hydrophobic residues and likely folds as an α-helix at the side of the α/β hydrolase domain as a regulatory lid, as suggested by CD and SAXS [35]. This region is post-translationally modified by SUMOylation at Arg14 [36] and by truncation in a *h*NDRG1 isoform found in different cell types [37,38].

Distinctively among other *h*NDRG proteins, *h*NDRG1 features a C-terminal sequence of 83 residues, rich in charged residues and featuring a three-times-repeated decapeptide GTRSRSHTSE. This region, here called *h*NDRG1*C (^311^GMGYMPSASMTRLMRSRTASGSSVTSLDGTRSRSHTSEGTRSRSHTSEGTRSRSHTSEAHLDITPNSGAAGNSAGPKSMEVSC^394^) binds to transition metal ions such as Ni(II) [30,39], Zn(II) and Cu(II) [40], as well as Mn(II) and Co(II) [40], and it is responsible for the conformational change observed in vitro for *h*NDRG1 upon interaction with lipids [35]. This sequence is modified both in vitro and in vivo through Ser-Thr phosphorylation performed by different kinases, such as calmoduline kinase-II, protein kinase A (PKA), serum- and glucocorticoid-induced kinase 1 (SGK-1) and glycogen synthase kinase 3 beta (GSK3beta) [41,42]. This post-translational modification likely regulates the biological functions of the *h*NDRG1, determining its cell-cycle-dependent protein localization and protein–protein interactions [38,43,44,45,46]. Phosphorylation of Ser330 and Thr346 by SGK1 is related to the suppression of the NF-ĸB signaling pathway and of the CXC cytokine production in pancreatic cancer cells [45]. The SGK1-mediated additional phosphorylation of Thr356 and Thr366, located—together with Thr346—in the decapeptide sequences and found in the liver, lung, spleen and skeletal muscle of mice, primes the further action of GSK3, which could then phosphorylate Ser342, Ser352 and Ser362 in the three repeated regions [42]. All these observations pinpoint an important role of this unique C-terminal region for the *h*NDRG1 characteristic cellular function.

In the present work, the structural, biochemical and biophysical characterization of the C-terminal 83-residues portion of *h*NDRG1 (here named *h*NDRG1*C) is reported. Analysis of the sequence assigned it to the family of the intrinsically disordered regions (IDRs). The protein was expressed and purified from *Escherichia coli*, and experiments of isothermal titration calorimetry, light scattering and circular dichroism were carried out to establish its metal-binding activity, as well as secondary and quaternary structure, which were then compared with those observed for the native *h*NDRG1. A thorough analysis of the spectroscopic fingerprint of ^1^H- and ^13^C-detected NMR spectra provided detailed information on the effect of pH and Ni(II) binding onto the structure of the protein backbone and side chains. The biophysical data were integrated with the analysis of the Ni(II)-induced expression, subcellular localization and oligomeric states of *h*NDRG1 natively expressed in two cell lines, one deriving from lung adenocarcinoma. The results are discussed considering the possible role of *h*NDRG1*C in the Ni(II)-driven lung cancer progression.

## 2. Materials and Methods

### 2.1. Gene Cloning

The gene coding for the full-length human NDRG1 (*h*NDRG1, Uniprot code: Q92597-1), 1197 bp, was commercially synthesized and cloned into the pEX-K4 subcloning vector by Eurofins, introducing the recognition sites for NdeI and BamHI restriction enzymes at the 5′ and 3′ positions, respectively. The *pEX-K4: hNDRG1* construct, purified from *Escherichia coli* XL10-Gold Ultracompetent Cells (Agilent, Santa Clara, CA, USA), was double-digested with the restriction enzymes NdeI and BamHI (Fermentas, Waltham, MA, USA). The DNA fragment, corresponding to the *hNDRG1* gene, was purified from a 1% (*w*/*v*) agarose gel and ligated, using T4 DNA ligase (Promega, Madison, WI, USA), into a modified *pET15b* expression vector (5.7 kb), previously digested with the same restriction enzymes, containing the sequence for a N-terminal Strep-tag (MASWSHPQFEKGAENLYFQGH) [47]. The purified construct *pET15b:hNDRG*1 was analyzed by restriction analysis and sequenced at both strands.

The C-terminal sequence of the protein (*h*NDRG1*C) was PCR-amplified from the *pET15b:hNDRG1* using the Easy-A High-Fidelity PCR Cloning Enzyme (Agilent, Santa Clara, CA, USA) and the primer pairs *h*NDRG_*C _F (5′-CAACATATGGGCTATATGCCGAGCG-3′)/T7-terminator_R (5′-GCTAGTTATTGCTCAGCGG-3′). The purified PCR product was double-digested with NdeI and BamHI restriction enzymes (Fermentas, Waltham, MA, USA) and cloned into the modified *pET15b* expression vector described above [47]. The resulting construct was analyzed by restriction analysis and sequenced on both strands. Subsequently, the obtained pET15b:hNDRG1*C was double-digested with NcoI and BamHI restriction enzymes (Fermentas, Waltham, MA, USA). The Strep-hNDRG1*C gene was cloned into the *pETZZ_1a* expression vector [48]. This construct coded for *h*NDRG1*C fused to a N-terminal His tag, an IgG-binding domain ZZ (ZZ-tag) and a Strep tag. The entire fusion protein could be excised by TEV cleavage between the StrepTag and *h*NDRG1*C, leaving a Gly-His dipeptide at the N-terminal region of the protein. Subsequent removal of the non-native His residue was performed by site-directed mutagenesis using the Quikchange mutagenesis kit (Agilent, Santa Clara, CA, USA) following the manufacturer’s instructions, to yield the wild-type sequence ^311^GMGYMPSASMTRLMRSRTASGSSVTSLDGTRSRSHTSEGTRSRSHTSEGTRSRSHTSEAHLDITPNSGAAGNSAGPKSMEVSC^394^.

### 2.2. Protein Expression and Purification

Large-scale expression of the full-length protein *h*NDRG1 followed transformation of *E. coli* BL21-CodonPlus (DE3) RiL competent cells (Agilent, Santa Clara, CA, USA) with *pET15b:hNDRG1*, and was achieved in 1 L of autoinduction medium [49] (10 g L^−1^ triptone, 5 g L^−1^ yeast extract, 5 g L^−1^ glycerol, 3.3 g L^−1^ (NH_4_)_2_SO_4_, 6.8 g L^−1^ KH_2_PO_4_, 7.1 g L^−1^ Na_2_HPO_4_, 0.120 g L^−1^ MgSO_4_, 0.5 g L^−1^ glucose, 2 g L^−1^ lactose), supplemented with 100 μg/mL of ampicillin and 34 µg/mL of chloramphenicol. The cells were grown at 37 °C for 4 h with vigorous stirring; then, the temperature was reduced to 26 °C and the expression was carried out for 18 h. The cells were harvested by centrifugation at 6000× *g* for 20 min at 4 °C and the cellular pellet was resuspended in 60 mL of the lysis buffer containing 50 mM Tris-HCl buffer pH 8, 150 mM NaCl, 1 mM EDTA, 5 mM DTT, 20 μg/mL DNaseI, 10 mM MgCl_2_. A protease inhibitor cocktail of 1 mM PMSF (Sigma-Aldrich, Saint Louis, MO, USA), 1 mM benzamidine hydrochloride (Acros Organics, Geel, Belgium), 5 µg/mL pepstatin A (VWR) was added. The bacterial cells were disrupted by two passages through a French pressure cell (SLM Aminco) at 20,000 pounds/square inch. The soluble fraction, obtained after removal of the precipitated material by centrifugation at 25,000× *g* for 20 min at 4 °C, was loaded onto a StrepTrap HP 5 mL column (GE Healthcare, Chicago, IL, USA), pre-equilibrated with 50 mM Tris-HCl pH 8, 150 mM NaCl, 1 mM EDTA and 5 mM DTT. The same buffer was used to wash out the unbound material until the absorbance at 280 nm returned to baseline. The protein was eluted with 30 mL of the elution buffer containing 2.5 mM D-desthiobiotin (IBA-Lifesciences, Göttingen, Germany), and the fractions containing *h*NDRG1 were combined and concentrated using 10-kDa cut-off membrane ultra-filtration units (Millipore, Burlington, MA, USA) before the final polishing step obtained on a Superdex 200 16/60 column equilibrated with 20 mM HEPES buffer at pH 7.5, containing 150 mM NaCl and 1 mM TCEP (working buffer). The final yield of the protein was 6–8 mg L^−1^ of initial culture. The purity of the purified protein was verified by SDS-PAGE using NuPAGE 4–12% Bis–tris acrylamide gels and the fractions containing *h*NDRG1 were concentrated and stored at −80 °C. The final protein concentration, referred to the monomeric form of the protein, was determined using as molar extinction coefficient at 280 nm the theoretical value 38,890 M^−1^ cm^−1^ calculated using the ProtParam website (https://web.expasy.org/protparam/, accessed on 17 June 2022).

Large-scale expression of the C-terminal peptide *h*NDRG1*C (residues 312–394) followed transformation of *E. coli* BL21-CodonPlus (DE3) RiL competent cells (Agilent, Santa Clara, CA, USA) with pETZZ1a:hNDRG1*C, and was achieved in 1–2 L of lisogeny broth (LB) at 37 °C, supplemented with 30 µg/mL kanamicin and 34 µg/mL chrolamphenicol with vigorous stirring. Protein expression was induced with 0.5 mM IPTG at OD_600_ = 0.6 and performed at 26 °C for 18 h. To produce labeled *h*NDRG1*C, bacterial cells were centrifuged before induction and transferred in one-fourth of the initial volume using 2x M9 minimal medium (1.26 g L^−1^ (NH_4_)_2_SO_4_, 12 g L^−1^ Na_2_HPO_4_, 6 g L^−1^ KH_2_PO_4_, 1 g L^−1^ NaCl, 4 g L^−1^ glucose, 0.240 g L^−1^ MgSO_4_), containing either ^15^N or ^15^N/^13^C isotopes, and induced with 0.5 mM IPTG [50,51], as above. Cells were harvested by centrifugation at 6000× *g* for 20 min at 4 °C, resuspended in 30 mL of 50 mM Tris HCl buffer pH 7.6, 300 mM NaCl, 20 mM imidazole, 20 μg/mL DNase I and 10 mM MgCl_2_; and a protease inhibitor cocktail of 1 mM PMSF (Sigma-Aldrich, Saint Louis, MO, USA), 1 mM benzamidine hydrochloride (Acros Organics, Geel, Belgium) and 5 µg/mL pepstatin A (VWR). The bacterial cells were disrupted by two passages through a French pressure cell (SLM Aminco, Urbana, IL, USA) at 20,000 pounds/square inch and the lysate was centrifugated at 76,000× *g* for 20 min at 4 °C. The clarified fraction was loaded onto a HisTrap HP 5 mL column (GE Healthcare, Chicago, IL, USA), pre-equilibrated with 25 mL of 50 mM TrisHCl pH 7.6 containing 300 mM NaCl and 20 mM imidazole. The column was washed with the same buffer until the baseline was stable and the protein was eluted by a linear gradient from 20 to 500 mM imidazole. The fractions containing the His-Tag/ZZ-tag/Strep-Tag fusion polypeptide were collected and incubated with a 1:50 protease:protein ratio with TEV protease for 3 h at room temperature. Subsequently, the protein was loaded onto a HiPrep 16/60 desalting column pre-equilibrated with 20 mM Tris-HCl pH 7.5, 2 mM EDTA and 1 mM DTT. *h*NDRG1*C was separated by the His-Tag/ZZ-tag/Strep-Tag peptide using a SP-sepharose cation exchange chromatography XK 16/10 column, equilibrated with the same buffer, and eluted by a linear gradient from 0 to 1 M NaCl. *h*NDRG1*C eluted at 240–280 mM NaCl. Fractions containing the protein were pooled, concentrated with 3 kDa MWCO Centricon ultra-filtration units (Millipore, Burlington, MA, USA) and further purified into a Superdex 75 XK 16/60 column (GE Healthcare, Chicago, IL, USA) equilibrated with the working buffer. Protein quantification was performed by amino acid analysis (Alphalyse, Odense, Denmark). The obtained extinction coefficient at 280 nm was 2450 cm^−1^ M^−1^. The final yield of the purified protein was 5–10 mg L^−1^ of initial culture.

The purity of both *h*NDRG1 and *h*NDRG1*C, as well as the molecular mass of the isolated variants under denaturing conditions, were estimated by SDS-PAGE using XCell SureLockTM Mini-Cell Electrophoresis System (Thermo Fisher Scientific, Waltham, MA, USA) apparatus and NuPAGE 4–12% or 12% Bis–tris acrylamide gels, stained using ProBlue Safe stain (Giotto Biotech, Firenze, Italy). The absence of any metal bound to the purified proteins was confirmed by ICP-ES, using a procedure previously described [52].

### 2.3. Isothermal Titration Calorimetry

Ni(II) binding to *h*NDRG1*C (90–130 µM) was investigated at 25 °C using a high-sensitivity VP-ITC microcalorimeter (MicroCal, Northhampton, MA, USA). The protein solution in the working buffer was loaded into the sample cell (cell volume = 1.4093 mL) and 29 injections of a 10 µL solution containing NiSO_4_ (2 mM) were added using a computer-controlled 310-µL microsyringe. Intervals of 300 s were applied between the injections to allow the system to reach thermal equilibrium after each addition. The heat of dilution was negligible, as verified by control experiments performed titrating Ni(II) over the buffer, under the same experimental conditions.

The data were processed using the Origin software package (MicroCal, Northhampton, MA, USA) and fitted using a nonlinear least-squares minimization algorithm to theoretical curves corresponding to different binding models. Δ*H* (reaction enthalpy change), *K_A_* (binding affinity constant), *n* (number of binding sites) and *N* (binding stoichiometry) were the fitting parameters. The *χ*^2^ parameter was used to establish the best fit. The reaction entropy was calculated using the equations: Δ*G* = -*RT* lnK_a_ (*R* =1.9872 cal mol^−1^ K^−1^, *T* = 298 K) and Δ*G* = Δ*H*—*T*Δ*S*. The values obtained for Δ*H* and Δ*S* are apparent, and include contributions not only from metal binding, but also from associated events such as protonation/deprotonation of the amino acid residues involved in the binding and consequent change in the buffer ionization state.

### 2.4. Circular Dichroism Spectroscopy

The secondary structures of *h*NDRG1 (30 µM) and *h*NDRG1*C (200 µM) were estimated by recording circular dichroism (CD) spectra in the working buffer using a JASCO J-810 spectropolarimeter flushed with N_2_ and a cuvette with 0.1 cm path length. Experiments were conducted in the absence and in the presence of different concentrations of NiSO_4_. Ten spectra were registered at 25 °C from 260 nm to 190 nm at 0.2 nm intervals and averaged to achieve an appropriate signal-to-noise-ratio. The secondary structure compositions of *h*NDRG1 and *h*NDRG1*C were quantitatively evaluated using Best Structure Selection (BeStSel) [53].

### 2.5. NMR Spectroscopy

NMR experiments were carried out using 0.2–0.3 mL samples of 0.8–1.0 mM purified U-^15^N or U-^13^C,^15^N *h*NDRG1*C in the working buffer at pH 6.5 containing 10% D_2_O, in 3-mm NMR tubes. Standard ^1^H-detected protein NMR spectra for the assignment of nuclei belonging to backbone [^1^H-^15^N BEST-TROSY, ^1^H-^13^C HSQC, HNCO, HNcaCO, HNCA, HNCACB, CBCAcoNH, HNcoCACB, HBHANH and HBHAcoNH], aliphatic side-chains [^1^H-^13^C HSQC, hCCH-TOCSY, HCcH-TOCSY and CcoNH] and aromatic side chains [2D ^1^H-^1^H TOCSY and hbCBcgcdHD (CBHD)] were collected at 298 K on a Bruker AVANCE NEO/III spectrometer operating at 28.2 T (1200.73 MHz ^1^H Larmor frequency) and equipped with a 3 mm triple-resonance inverse TCI z-gradient cryoprobe. ^13^C-detected NMR spectra (CON, hCACO and hCBCACO) [54,55,56] were acquired at 298 K using a 16.4 T Bruker AVANCE NEO spectrometer operating at 16.4 T (700.06 MHz ^1^H Larmor frequency), equipped with a 3 mm TXO cryoprobe optimized for ^13^C direct detection. Proton chemical shifts were referenced to 2,2-dimethyl-2-silapentane-5-sulfonic acid sodium salt (DSS), while the ^13^C and ^15^N chemical shifts were referenced indirectly to DSS, using the ratios of the gyromagnetic constants. Table S1 reports the NMR spectra acquisition parameters.

All NMR spectra were processed using NMRpipe [57] and the SMILE (Sparse Multidimensional Iterative Lineshape-Enhanced) [58] reconstruction algorithm plug-in module implemented in NMRpipe, for both non-uniformly sampled (NUS) and conventional NMR spectra. Table S1 reports the details of all acquired NMR spectra. Analysis and assignments of the 2D and 3D data sets were carried out using NMRFAM-SPARKY [59] and POKY. [60] The assignment process was facilitated by using the PINE [61,62] server for initial automated assignments before completing the assignments manually. LACS (Linear Analysis of Chemical Shifts) [63] was used to validate the final assignment. The assignment was deposited in the Biological Magnetic Resonance Bank (BMRB) with the accession code 50803.

^1^H-NMR experiments tailored for the identification of hyperfine shifted and fast relaxing signals [64] were performed on an AVANCE 600 Bruker NMR spectrometer equipped with a room-temperature 5 mm ^1^H selective probe and operating at 600.13 MHz ^1^H Larmor frequency, without gradients. Spectra were collected using a short ^1^H pulse (3 µs, corresponding to a ca. 35° pulse), in order to excite the large spectral window (640 kHz) needed for the experiments. A 200 ms presaturation pulse was applied to suppress the water signal. A total of 32 K data points were acquired over 46 ms. The number of scans acquired ranged from 200 K to 400 K, corresponding to 18–36 h of experimental time. Prior to Fourier transform, FIDs were multiplied by a cosine-square weighting function followed by a 40 Hz Lorentzian line broadening. Phase and baseline correction were performed manually.

### 2.6. Light Scattering

The oligomeric properties and the hydrodynamic radii of *h*NDRG1 and *h*NDRG1*C in the absence and in the presence of Ni(II) were determined combining size-exclusion chromatography (SEC) with multiple-angle light scattering (MALS) and quasi-elastic light scattering (QELS). *h*NDRG1 (300 µL, 140 µM) and *h*NDRG1*C (300 µL, 1.2 mM) in the working buffer were loaded onto a Superdex 200 10/300 GL column (GE Healthcare, Chicago, IL, USA) (*h*NDRG1) or a Superdex 75 10/300 GL column (GE Healthcare, Chicago, IL, USA) (*h*NDRG1*C) equilibrated with the same buffer, in the absence or in the presence of equimolar concentrations of Ni(II). NiSO_4_ (28 µM for *h*NDRG1 and 240 µM for *h*NDRG1*C) was also added to the working buffer for the experiments in the presence of Ni(II). Elution was carried out at room temperature with a flow rate of 0.5 mL/min. The column was connected downstream to a multiple-angle laser light (690 nm)-scattering DAWN EOS photometer (Wyatt Technology) and to a quasi-elastic light-scattering apparatus (Wyatt QELS). The used value for the specific refractive index increment (dn/dc) was 0.185 mL/g [65]. The value of 1.331 for the solvent refractive index was determined using the refractive index detector. The latter was used also to determine the concentration of the protein while eluting from the chromatographic column. The weight-average molecular masses were determined from MALS measurements across the entire elution profile, in intervals of 0.2 s, using the ASTRA software (Wyatt Technology). A Rayleigh–Debye–Gans light-scattering model was used to determine the molecular weight (MW), using a Zimm plot. The uncertainties on MW are a measure of the statistical consistency of the MALS data, obtained combining the standard deviations calculated for each slice in the analyzed peaks. Data analysis was performed using Astra version 5.3.4 following the manufacturer’s instructions.

### 2.7. Cultures and Cellular Treatments

Human A549 cell line derived from lung carcinoma was a gift from Dr A. Tesei (Istituto Scientifico Romagnolo per lo Studio e la Cura dei Tumori, Meldola, Italy). HeLa cells were obtained from Istituto Ortopedico Rizzoli, Bologna Italy. Cells were cultured in Dulbecco’s Modified Eagle Medium (DMEM) GlutaMAX (Gibco, ThermoFisher Scientific, Monza, Italy) supplemented with 10% of heat-inactivated fetal bovine serum (FBS, Gibco). Cells were maintained in a humidified atmosphere with 5% CO_2_ at 37 °C and subcultured twice a week. Where indicated, cells were treated with NiSO_4_.

### 2.8. Preparation of Protein Extracts

Total lysates were prepared in SDS lysis buffer (20 mM Tris-HCl, pH 7.5, 1% SDS, 1 mM Na_3_VO_4_, 1 mM PMSF, 5% β-mercaptoethanol and protease inhibitors). Nuclear extracts were prepared as follows: cells were trypsinized, collected and resuspended in hypotonic buffer (10 mM Tris-HCl pH 7.8, 5 mM MgCl_2_). Then, 0.2% Triton X-100 was added. Cells were sheared through a 22-G needle. Nuclei were recovered by centrifugation and lysed in 20 mM Tris-HCl 7.0, 1% NP-40, 150 mM NaCl, 10% glycerol, 10 mM EDTA, 20 mM NAF, 5 mM Na_4_P_2_O_7_, 1 mM Na_3_VO_4_, 1 mM PMSF and protease inhibitors and cleared by centrifugation. Protein amount was evaluated by Bradford colorimetric assay. Equal amounts of protein lysates were resolved by SDS-PAGE. Nondenaturing conditions were maintained by resuspending lysates in native sample buffer (0.3 M Tris-HCl pH 6.8, 0.03% bromophenol blue and 50% glycerol); denaturing conditions were achieved after boiling samples resuspended in full Laemli sample buffer (0.3 M Tris-HCl pH 6.8, 0.03% bromophenol blue, 9% sodium dodecyl sulfate (SDS), 9% β-mercaptoethanol, 50% glycerol). Samples were transferred to nitrocellulose membrane overnight at 4 °C. Incubation with primary antibodies was performed for the indicated time. Bands were revealed using the Amersham ECL detection system and analyzed with ImageJ (National Institute of Health, Bethesda, MD, USA). Purity of nuclei was analyzed by immunoblot detection of β-tubulin. Antibodies used were anti-NDRG1 (1:500), anti-Actin (1:1000), anti-Lamin A/C (1:200), all from Santa Cruz (Santa Cruz Biotechnology, DBA Italia SRL, Segrate, Italy); anti-β-Catenin (1:3000), α-tubulin (1:2000) and anti-GAPDH (1:8000) from Merck (Merck Life Science S.r.l., Milan, Italy); anti-p21 (1:1000) (Thermo-Fisher Scientific).

## 3. Results

### 3.1. Analysis of Protein Disorder

**Figure 1:**
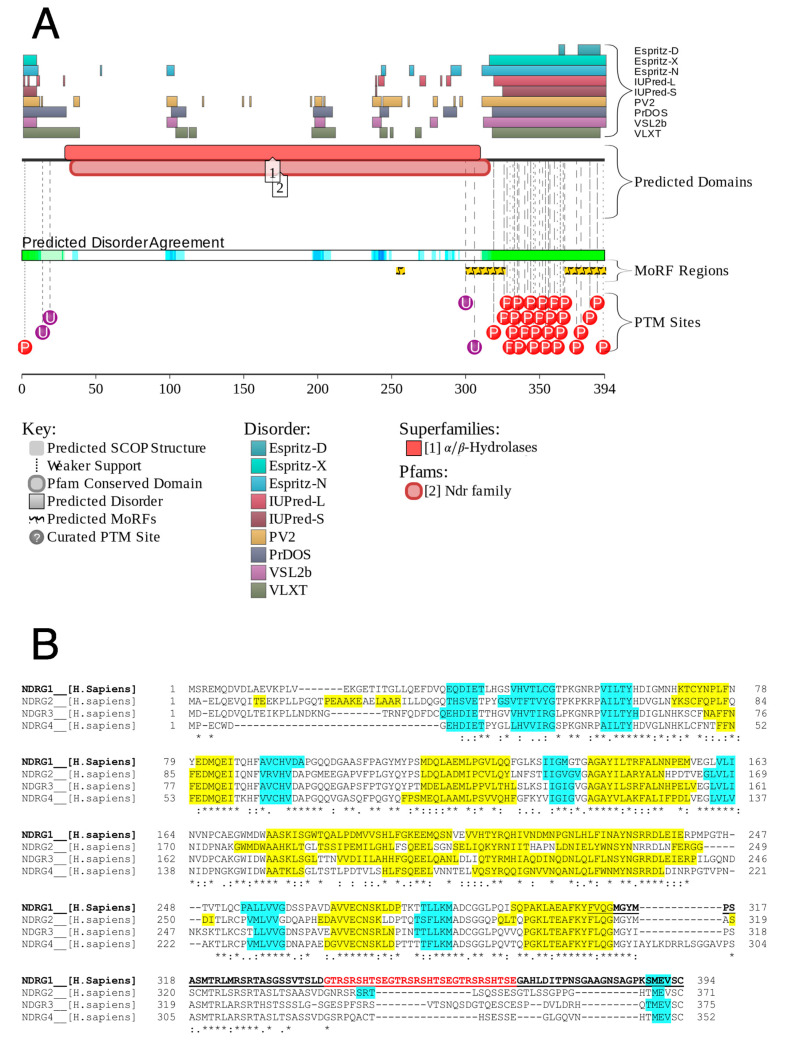
Analysis of the sequence of *h*NDRG1. (**A**) Disordered regions of the sequence of *h*NDRG1 as predicted by the D2P2 server (http://d2p2.pro/, accessed on 29 January 2021). The predicted disordered regions (top), folded domains (middle), and disorder consensus (bottom) are indicated by bars over the residue numbers. The sites with predicted post-translational modifications are also indicated. (**B**) Prediction of the secondary structure content of *h*NDRG1 using the software JPred [67]: predicted a-helices are indicated in yellow, b-strands are in cyan. *h*NDRG1*C sequence is underlined and the repeated decapeptide is shown as red text.

Analysis of the canonical sequence of *h*NDRG1 (UniProt accession number Q92597) using disorder predictors in the D2P2 database [66] highlighted a differentiation in disorder content along the protein sequence (Figure 1A) that concerns the three domains previously reported [35]: indeed, disorder is prevalent in the short N-terminal sequence (residues 1–30) and in the unique C-terminal domain of 83 residues (*h*NDRG1*C, residues 312–394), which contains several predicted phosphorylation sites, as well as a three-times-repeated His-containing decapeptide with Ni(II)-binding properties (Figure 1B) [30,39]. On the other hand, the α/β hydrolase domain (residues 30–312) is predicted to be well-folded (Figure 1B). 

According to the BioGRID database (https://thebiogrid.org, accessed on 29 January 2021), *h*NDRG1 interacts with as many as 140 proteins in the cell [68]. Consistently, a visualization of the interactivity of *h*NDRG1 by the STRING computational platform [69], which produces a network of predicted associations for a particular group of proteins, shows that this protein is part of a massive interactome (Figure S1A). The intrinsically disordered nature of the C-terminal region might be indicative of the observed promiscuity of *h*NDRG1, reported to be involved in a plethora of different cellular metabolisms [15]. This observation is supported by a prediction of two molecular recognition sequences (MORF, Figure 1A) [70] at the beginning and at the end of the protein C-terminus, as well as by the output of the Anchor software [71], which identifies a long disordered-based interaction sequence that includes *h*NDRG1*C (residues 308–394, Figure 1B). Coherently, the charge–hydropathy (CH) plot [72], which analyzes the protein properties in terms of the mean net absolute charge vs. mean hydrophobicity, shows that while the full-length protein falls in the region of the plot typical of polypeptides with a well-folded behavior, *h*NDRG1*C resides in the disordered region of the plot, confirming its propensity for an intrinsically disordered behavior (Figure S1B).

### 3.2. Protein Expression and Purification

The canonical sequence coding for *h*NDRG1 was obtained by retrotranslating its amino acid sequence and cloned into a modified *pET15b* expression vector. The obtained construct was used to overproduce a N-terminal Strep-tagged protein (45.28 kDa) in *E. coli* BL21-CodonPlus (DE3) RiL competent cells using an autoinduction medium enriched with glucose and lactose. The expressed polypeptide accumulated in the soluble fraction of the cellular extract and was purified using a Strep-tactin affinity column, followed by size-exclusion chromatography. The identity and purity of the protein was confirmed by gel electrophoresis (Figure S2A) and by mass spectrometry, which evidenced the cleavage of the N-terminal methionine after protein production. In the SDS-PAGE, the presence of a band at ca. 85–90 kDa, coexisting with that expected at 45 kDa and corresponding to the protein monomer, suggests a possible dimerization of the protein sample (Figure S2A). This is sometimes visible in SDS-PAGE, when denaturing conditions used (in this case, heating the sample at 90 °C for 3 min) are not sufficient to fully dissociate the dimer.

The nucleotide sequence coding for *h*NDRG1*C was initially cloned into a modified *pET15b* vector, but the expression of the Strep-tagged protein was insignificant. Thus, a different expression approach, involving the N-terminal tagging of *h*NDRG1*C with the modified immunoglobulin-binding domain of protein A of *Staphylococcus aureus* (ZZ-tag), fused with a His-rich sequence at the N-terminus, was applied [48]. This tag allowed for the improvement of yields, solubility and conformational stability of the expressed protein. The obtained construct was used to overproduce His-ZZ-Strep-*h*NDRG1*C (27.98 kDa) in *E. coli* BL21-CodonPlus (DE3) RiL cells by induction with IPTG. The polypeptide was purified from the soluble fraction of the cellular extract with a Ni(II)-based affinity chromatography, followed by TEV protease cleavage and by cation-exchange chromatography. High-purity *h*NDRG1*C (8.83 kDa) was obtained using size-exclusion chromatography (SEC). The identity and purity of the protein were confirmed by gel electrophoresis (Figure S2B) and by mass spectrometry.

### 3.3. Isothermal Titration Calorimetry

**Figure 2:**
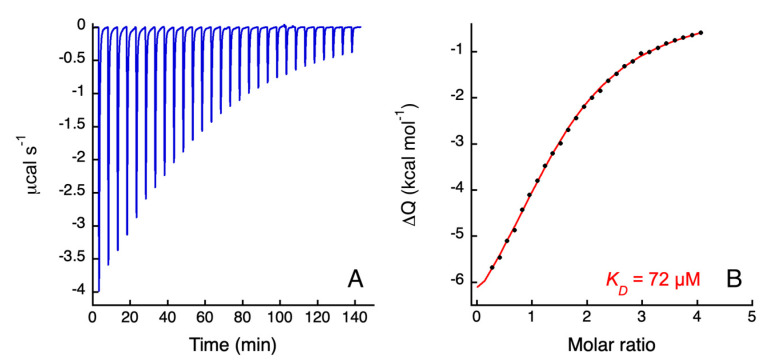
ITC titration data for the binding of NiSO_4_ to *h*NDRG1*C. (**A**) Raw titration data represent the thermal effect of 27 × 10 µL injections of Ni(II) onto or *h*NDRG1*C solution at pH 7.5. (**B**) Normalized heat of reaction data for the binding events of Ni(II) to *h*NDRG1*C were obtained integrating the raw data. The solid line represents the best fit of the integrated data, obtained by a nonlinear least-squares procedure, as described in the text. The calculated dissociation constant is indicated.

Titration of Ni(II) on *h*NDRG1*C (Figure 2A) produces a binding isotherm with a single inflection point, which was fitted using a single site model. The binding parameters, obtained from the fit of four binding isotherms (Figure 2B) and by averaging the results of the fits, indicate that the protein presents a single binding event (*n* = 1.4 ± 0.5) with affinity in the micromolar range (*K_A_* = 1.4 ± 0.3 × 10^4^*; K_D_* = 70 ± 1 µM) and enthalpically driven (ΔH = −9 ± 6 kcal mol^−1^ and ΔS = −19 ± 13 cal mol^−1^ K^−1^).

### 3.4. Circular Dichroism

**Figure 3:**
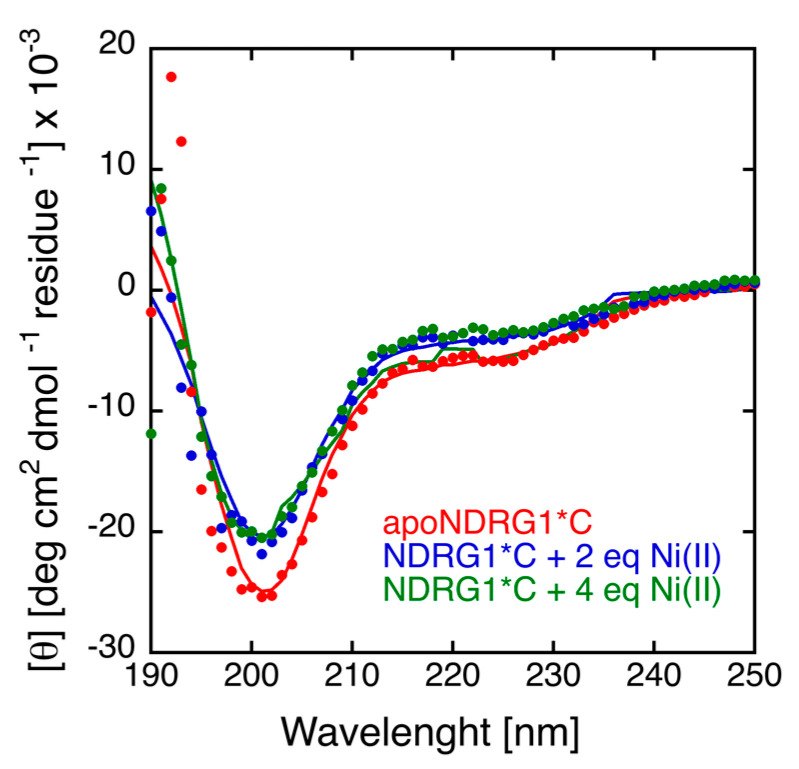
Far-UV CD spectra of *h*NDRG1*C in the absence and in the presence of increasing concentrations of Ni(II).

The CD spectrum of *h*NDRG1*C is typical of an intrinsically disordered protein, with a pronounced negative peak around 198 nm and a quantitative analysis of the spectrum confirming the low amount of secondary structure, with 6.6% α-helices, 26.3% β-strands and 67% unordered (Figure 3). The addition of Ni(II) does not significantly influence the protein secondary structure (Figure 3). The CD spectrum of *h*NDRG1 in the presence and in the absence of Ni(II), shown in Figure S3 for comparison with the one of *h*NDRG1*C, is very similar to that reported previously [35], independently from the presence of Ni(II) ions. Quantitative evaluation of secondary structures, using BestSel, indicated a marked prevalence of α-helical content (25%) and a minor number of β-strands (19%), well in accordance with the secondary structure content extracted from the crystal structure (25% α-helix and 12.5% β-strands) [35].

### 3.5. NMR Spectroscopy on hNDRG1*C

In order to establish the molecular structural details of *h*NDRG1*C in solution at the atomic/molecular level and the effects of Ni(II) binding, high-resolution nuclear magnetic resonance spectroscopy (NMR) was extensively applied. The full signal assignment was first achieved at pH 6.5, followed by the analysis of the effect of solution pH on the spectra and finally by the investigation of the modifications induced by Ni(II) binding at pH 7.5.

#### 3.5.1. ^1^H, ^13^C and ^15^N Signal Assignment

Two-dimensional and three-dimensional high-resolution solution NMR spectra of *h*NDRG1*C were acquired using data recorded at 1.2 GHz ^1^H Larmor frequency, with the aim of assigning the backbone and side-chain signals of the protein. In the following description, the residues are numbered according to the sequence of the full *h*NDRG1 protein, from Gly311 to Cys394. Initial attempts to record ^1^H,^15^N HSQC spectra at the pH of the working buffer (7.5) resulted in a significantly lower number of signals than expected, suggesting the presence of exchange phenomena involving the amide NH protons at this pH. On the other hand, lowering the pH at 6.5 resulted in a significant improvement of the spectra; therefore, the NMR signal assignment was initially carried out at pH 6.5.

**Figure 4:**
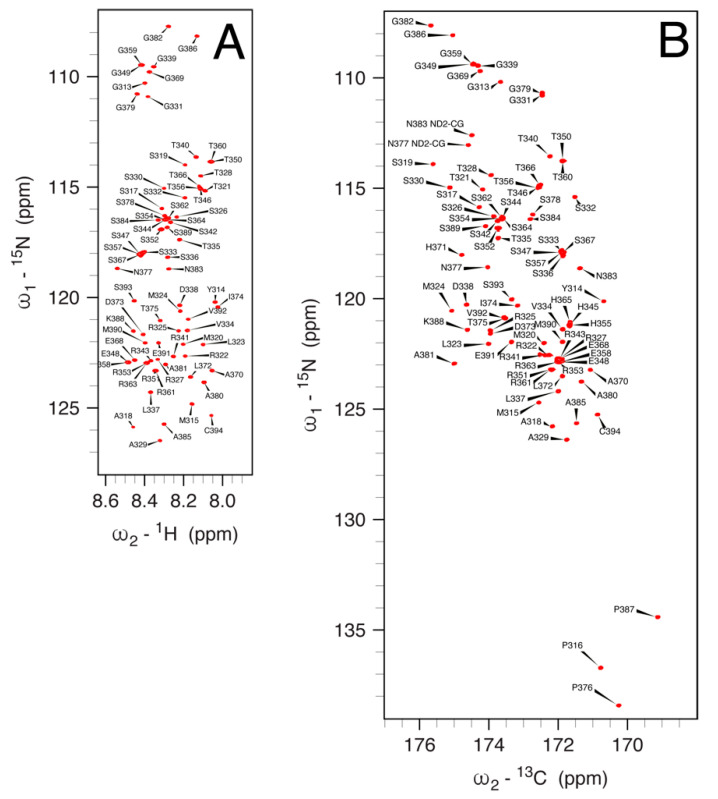
(**A**) 1.2 GHz (28.2 T) 2D ^1^H,^15^N BEST-TROSY spectrum and (**B**) 700 MHz (16.4 T) 2D CON spectrum obtained by ^13^C direct detection, acquired at 298 K on samples of ^13^C,^15^N labeled *h*NDRG1-C, at pH 6.5. In the CON spectrum, the resonances are labeled according to the ^15^N frequency. Assigned peaks are labeled.

The BEST-TROSY ^1^H-^15^N HSQC spectrum of hNDRG1*C at pH 6.5 (Figure 4A) features a narrow ^1^H chemical shift dispersion (8.0–8.6 ppm) typically seen for intrinsically disordered proteins (IDPs). Outside this region, the signals of amide NH_2_ protons of the side chains of the two asparagine residues Asn377 and Asn383 could also be detected. A total of 76 amide NH signals were assigned in the ^1^H-^15^N HSQC spectrum. The N-terminal is not observed because of the fast exchange with water, while the NH resonance of Met312 is not detected probably because its NMR signal is broadened beyond detection due to conformational exchange phenomena occurring with rates comparable to the frequency differences among the different conformers. In addition, Pro316, Pro376 and Pro387 are not observable in the ^1^H,^15^N HSQC spectrum, but the signals of CB and CG of these residues were obtained using triple-resonance experiments; the conformation of these residues was determined by calculating the difference between proline CA and CG chemical shifts [73,74], which indicated that all proline residues are linked to the preceding amino acid by a peptide bond in the *trans* conformation. The ^13^C chemical shift of the CB nucleus of Cys394, the last protein residue in the sequence and the only cysteine residue in the sequence, is 29.06 ppm; considering that this value depends on the redox state of the terminal S atom, being <32 ppm for the reduced thiol state and >35 ppm for the oxidized disulfide state [75], it can be concluded that Cys394 is in the Cys-SH state, preventing the formation of higher-order aggregates by disulfide bridges; this is consistent with the observation that the signals display peak widths and intensities that are invariant upon dilution, as well as with the results of light-scattering measurements.

**Figure 5:**
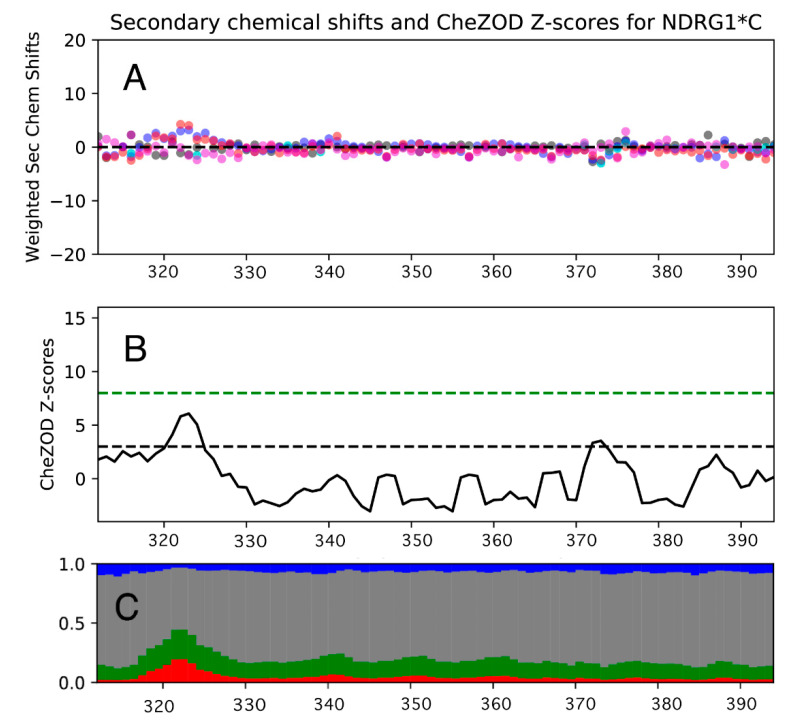
(**A**) Weighted secondary chemical shifts, (**B**) CheZOD Z-scores and (**C**) stacked bar plot of CheSPI populations of “extended” (blue), “helical” (red), “turn” (green) and “non-folded” (grey) local structures calculated for *h*NDRG1*C.

The essential absence of significant secondary structures in *h*NDRG1*C was confirmed by the inspection of the deviations of the chemical shifts of backbone nuclei from their predicted random coil chemical shifts (RCCS), carried out using CheZOD [76] and CheSPI [77], specific algorithms to quantify the statistical composition of structural states in IDPs (http://www.protein-nmr.org, accessed on 25 February 2022). This analysis indicated that NDRG1*C is largely unfolded, with the notable exception of the ^318^ASMTRLMRS^327^R region near the N-terminus, which features a small α-helix propensity, consistent with a transient helical character (Figure 5).

**Figure 6:**
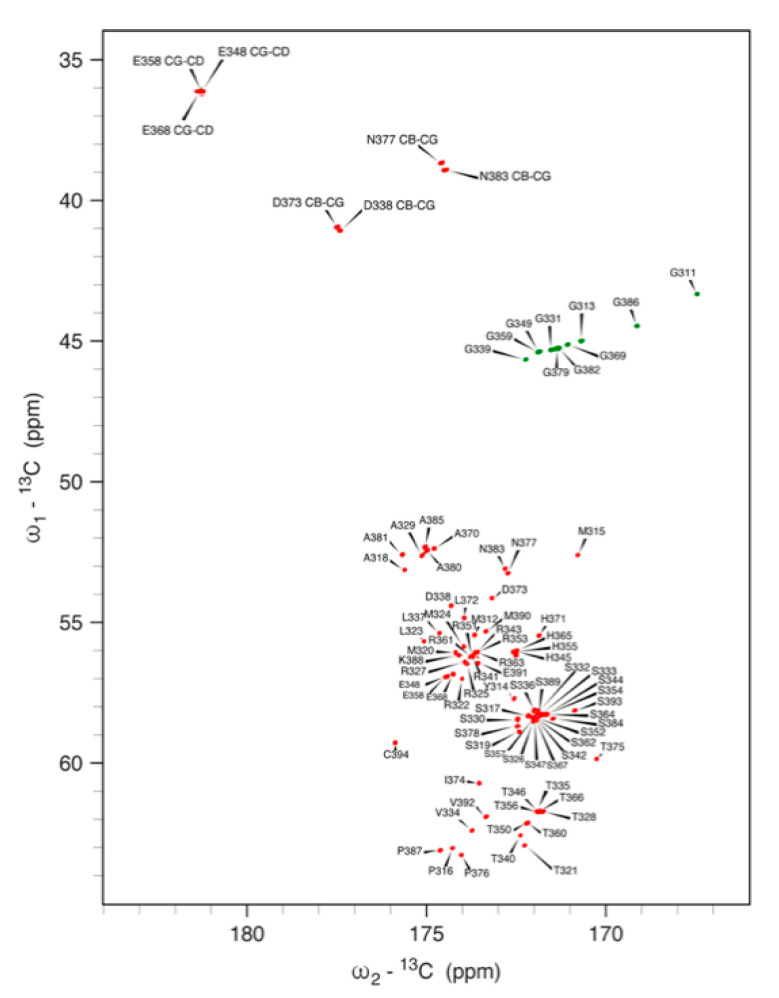
700 MHz (16.4 T) 2D CACO spectrum obtained by ^13^C direct detection, acquired at 298 K on samples of ^13^C,^15^N labeled *h*NDRG1-C, at pH 6.5. The resonances are labeled according to the ^13^C frequencies of the CO and CA nuclei of each amino acid, except for the signals for the side chains of Asp and Glu residues, for which the CB-CG and CG-CD are explicitly indicated.

Notably, the peptide NH signals for all histidine residues are missing from the ^1^H,^15^N HSQC spectrum: it has been shown that amide proton exchange with water significantly increases in the presence of a protonated histidine imidazole ring [78], suggesting that the side chains of His345, His355, His365 and His371 are in the imidazolium form at the solution pH of 6.5. On the other hand, the ^13^C-detected CON spectrum, which correlates the peptide ^15^N nucleus of each residue to the carbonyl ^13^C nucleus of the preceding amino acid independently of the amide ^1^H exchange phenomena, allows the observation of the ^15^N signals of all histidines, together with those of Pro316, Pro376 and Pro387 (Figure 4B). The assignment of the side-chain ^13^C nuclei of each residue, performed using triple-resonance experiments, allowed for the full assignment of the ^13^C-detected CACO (Figure 6) spectrum, which additionally revealed the signals for the side-chain carboxyl and amide carbons of Asp338, Asp373, Glu348, Glu358, Glu368, Asn377 and Asn383; for the latter two residues, the side-chain amide ^15^N nuclei could also be assigned in the CON spectrum (Figure 4B).

Considering the potential importance of the histidine residues in Ni(II) binding, a special effort was dedicated to the full assignment of the side chains of these four amino acids using a combination of NMR spectra. The general scheme for this task is illustrated in Figure S4, and the full assignment is reported in Table 1. Considering the characteristic chemical shifts of the CD2 and CE1 nuclei for the histidine imidazole ring in the neutral (113.6 and 135.5 ppm) and doubly protonated cationic (119.4 and 136.3 ppm) states [79,80,81], the chemical shift values for all histidine residues (CE1 ~138 ppm and CD2 ~120 ppm) support the doubly protonated state of all imidazole rings at pH 6.5. This was confirmed by the patterns observed in the 2J ^1^H,^15^N-HMQC spectra, which revealed chemical shifts for ND1 and NE1 nuclei (in the 180–210 ppm range), typical for doubly protonated His residues, [79,80,81] and is consistent with a low Ni(II)-binding capability of *h*NDRG1*C at pH 6.5, due to this unfavorable protonation state of the His residues.

#### 3.5.2. Effect of pH on the NMR Spectra

Considering that Ni(II) binding would be more physiologically significant to explore at pH higher than 6.5, the effect of pH on the NMR spectra of *h*NDRG1*C was monitored by recording the ^1^H,^15^N HSQC, CON and CACO fingerprint spectra in the 6.5–7.5 pH range with steps of 0.25 pH units, allowing us to assign the NMR signals observed at pH 7.5. Similarly, the ^1^H,^13^C HSQC in the aromatic region was monitored in the same pH range to assign the observed signals to the non-exchangeable protons of the histidine side chains.

The intrinsically disordered behavior of the protein is not affected by pH in the 6.5–7.5 interval. The ^1^H,^15^N HSQC spectrum at pH 7.5 is essentially obliterated, consistently with the increased rate of hydrogen exchange (HX) at higher pH, except for signals related to Tyr314, Met315, Leu323, Asp338, Ala370, Leu372, Asp373, Ile374, Thr375, Gly386, Lys388, Glu391, Val392, Ser393 and Cys394; while the chemical shifts of most of these signals are not affected by the pH change, the signals of Leu372 and Asp373 are clearly perturbed, which is a phenomenon that can be rationalized by considering that in the protein sequence, they are in the vicinity of His371, a residue that could be involved in a protonation/deprotonation event in the explored pH range.

This observation reflects different hydrogen-exchange (HX) behaviors along the protein backbone and could be used to gain information about the structure and conformational dynamics of *h*NDRG1*C. The HX rate is determined by a number of factors, including solvent shielding caused by the presence of folded segments, H-bond formation, amino acid sequence composition, temperature and ionic strength [82]. The presence of structural elements and H-bond formation will slow HX, and this is expressed by the so-called protection factor (PF = *k*_intr_/*k*_obs_), namely the ratio between the intrinsic HX rate in a random coil model (*k*_intr_) and the HX rate measured for the protein (*k*_obs_) [83]. The values of *k*_intr_, which depend on the protein sequence, are normally not available, but can be calculated by considering the identity of the side chains bracketing each of the amide hydrogens in the sequence [84,85] using SPHERE (https://protocol.fccc.edu/research/labs/roder/sphere/sphere.html, accessed on 25 February 2022). Another crucial parameter that influences the HX rates is the local electrostatic potential at all backbone amide positions along the chain [82]. Plots of (i) the amide NH signal intensities at pH 6.5, (ii) the k_intr_ values calculated using SPHERE, as well as (iii) the electrostatic potential and (iv) the protection factor computed using a recently proposed approach [82], all calculated at pH 6.5 (Figure S5), suggest that the observed smaller effect of pH on the intensity of signals at the C-terminal portion of the protein vs. the N-terminal region is not due to an increased propensity towards a more structured ensemble, but rather to a decrease in the positive electrostatic potential, observed for the first ca. 65–70 residues in the sequence, to smaller and even negative values in the last 15–20 residues of the sequence. The consequence of this trend is a decrease in the local hydroxide ion concentration in the region of the protein that features a smaller electrostatic potential, which in turn induces a decreased HX rate and an increase in the signal intensity at higher pH.

On the other hand, in the CON and CACO spectra, no significant modification of the signals intensities is observed in the explored pH range, consistently with the independence of these signals on amide proton solvent exchange phenomena. In these spectra, the largest chemical shift perturbations affect the signals of the histidine residues, indicating that the deprotonation events occurring upon raising the pH from 6.5 to 7.5 only have local effects, without significant modification of the overall conformational space occupied by the protein. To analyze the effect of pH on the histidine side chains, the dependence of the ^1^H chemical shifts of the HE1 signals in the ^13^C HSQC spectra of *h*NDRG1*C in the aromatic region as a function of pH in the 6.5–7.5 range was investigated. The corresponding pK_a_ values for the four histidine residues were obtained from simultaneous nonlinear fits of the chemical shifts of HE1 and HD2 protons of each imidazole ring to the following one-ionization Equation (1):

(1) $$\delta_{obs}=\frac{\left[H^{+}\right]\cdot \delta_{HisH}+K\cdot \delta_{His}}{\left[H^{+}\right]+K}$$

where *d_obs_* is the observed experimental chemical shift, *d_HisH_* and *d_His_* are the chemical shifts of the protonated and neutral forms of the histidine imidazole, and *K* is the dissociation constant for the ionization equilibrium. The estimated pK_a_ values (Figure S6) are similar for all histidines (6.65 for His335 and His345, 6.72 for His355, and 6.84 for His371), with a significant fraction (10–20%) of protonated states still maintained at pH 7.5 in all cases.

#### 3.5.3. Ni(II) Binding to NDRG1*C by NMR Spectroscopy

**Figure 7:**
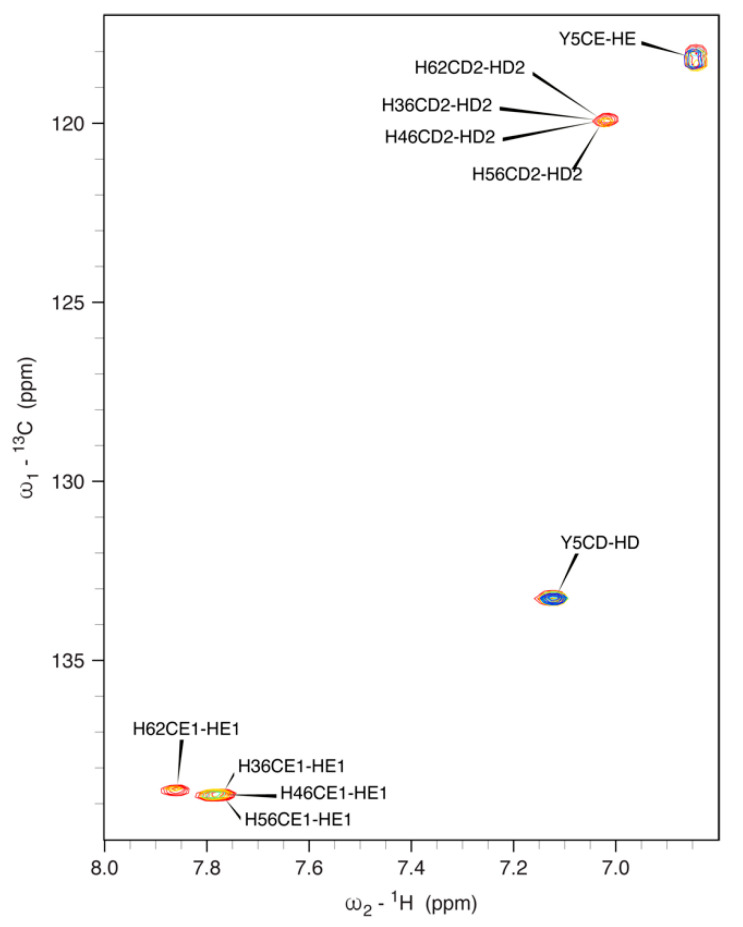
A 700 MHz (16.4 T) ^1^H,^13^C HSQC spectrum of *h*NDRG1*C in the aromatic region, highlighting the signals for the side-chain imidazole rings of His345, His355, His365 and His371, as well as Tyr314, at pH 7.5, as a function of incremental addition of Ni(II) (red: 0 eq; orange: 1 eq; cyan: 2 eq; blue: 3 eq).

**Figure 8:**
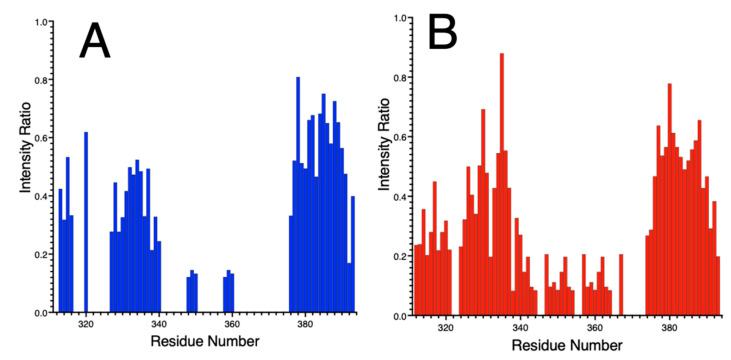
Intensity ratios of the peaks in the (**A**) CON spectrum and (**B**) CACO spectrum obtained at 700 MHz (16.4 T) by ^13^C direct detection at pH 7.5 in the absence and presence of 3 eq. Ni(II).

The obtained assignment of NMR spectra at pH 7.5 was then used to monitor the effects of Ni(II) binding onto *h*NDRG1*C. The ^1^H-^13^C HSQC spectra in the aromatic region, where the signals of the CE1-HE1 and CD2-HD2 could be monitored as a function of the Ni(II)/protein ratio in the 0 to 3 equivalents (an upper limit known from calorimetry to saturate the binding equilibrium), indicated the progressive and concomitant disappearance of the signals of His371, His345, His355 and His365 without any detectable selectivity (Figure 7). The CON (Figure S7) and CACO (Figure S8) spectra further revealed that several additional signals disappear upon adding Ni(II) to the polypeptide solution, while those signals that are still visible do not modify their chemical shift. This phenomenon indicates that the protein does not undergo any observable change in the backbone folding upon Ni(II) binding. A large portion of the signals centered around the repeated decapeptide decrease their intensity upon Ni(II) binding, and in particular the N, CO and CA signals of all histidine residues are absent in the spectrum of the Ni-bound protein (Figure 8). Characteristically, the N, CO and CA signals of the residues that precede and follow the histidines are also canceled, suggesting the presence of bound paramagnetic high-spin S = 1 Ni(II) d^8^ ions hexacoordinated in (pseudo)octahedral geometry. In addition, the signals relating the side-chain carbonyl C atoms of Asp338, Glu348, Glu358, Glu368 and Asn377 disappear from the CON and CACO spectra, while the corresponding signals for Asn383 are not affected by Ni(II) binding. Finally, the N, CO and CA signals of the C-terminal Cys394 are completely obliterated upon Ni(II) binding, which is an indication that its thiolate group and/or the carboxylate C-terminus are involved in Ni(II) binding. Overall, the picture that can be drawn from the NMR spectra analysis indicates the involvement of the side chains of Asp338, His345, Glu348, His355, Glu358, His365, Glu368, His371, Asp373, Asn377 and Cys394 in the uptake of Ni(II) by *h*NDRG1*C. In the following sequence, the residues observed to bind Ni(II) are shown in red, while the decapeptides are underlined:

^311^GMGYMPSASMTRLMRSRTASGSSVTSLDGTRSRSHTSEGTRSRSHTSEGTRSRSHTSEGAHLDITPNSGAAGNSAGPKSMEVSC^394^.

#### 3.5.4. Effects of Ni(II) Binding by Paramagnetic NMR

**Figure 9:**
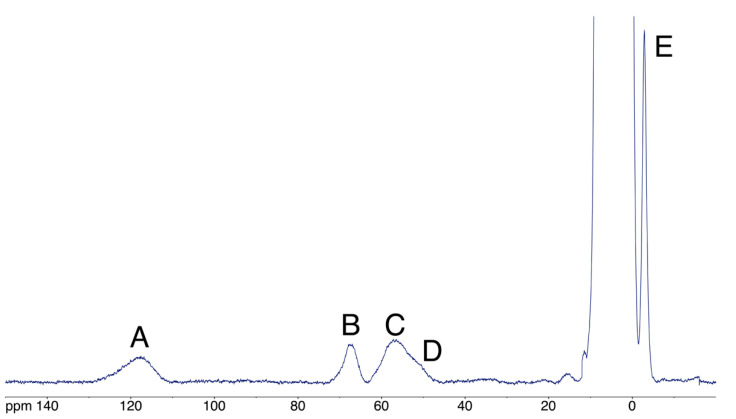
^1^H NMR spectrum of NDRG1*C at pH 7.5 and 298 K in the presence of 4 equiv. Ni(II). Signals A, B, C, D and E are relative to nuclei sensing the hyperfine shift (contact and pseudo-contact) due to the presence of the paramagnetic Ni(II) S = 1 ion bound to *h*NDRG1-C.

The interaction between *h*NDRG1*C and Ni(II) was then investigated using ^1^H NMR spectra tailored for the observation of signals of residues bound to the Ni(II) center and affected by its paramagnetism. The spectrum of *h*NDRG1*C in the presence of four equivalents of Ni(II) at pH 7.5 and 298 K, (Figure 9) contains five hyperfine-shifted signals (A-E) in the range from +130 to −10 ppm, outside the diamagnetic region, arising from contact and pseudo-contact shifts involving high-spin (S = 1) Ni(II) centers. The chemical shifts, line widths and the Curie-type temperature dependence of the chemical shifts are consistent with the presence of a single paramagnetic Ni(II) center with S = 1 in octahedral coordination [64,86]. Signal B, at 68 ppm, disappears in D_2_O, indicating that it belongs to an exchangeable proton; these features suggest that it belongs to either Hε2 or Hδ1 of Ni-bound histidine residues [64,86,87,88]. Signals C and D belong to non-exchangeable protons; their chemical shift is typical for imidazole Hε1 and Hδ2 histidine protons [64,86,87,88]. Signal A disappears by lowering the temperature to 288 K: its chemical shift is consistent with Hβ protons of Ni(II)-bound cysteine residues [64,89,90], suggesting the involvement of the C-terminal Cys394 thiol. The fact that its intensity varies with temperature is interpreted as indicating the presence of exchange phenomena by which Ni(II) is binding to different sites with equilibria that shift according to the available kinetic energy of the system.

### 3.6. Light Scattering

**Figure 10:**
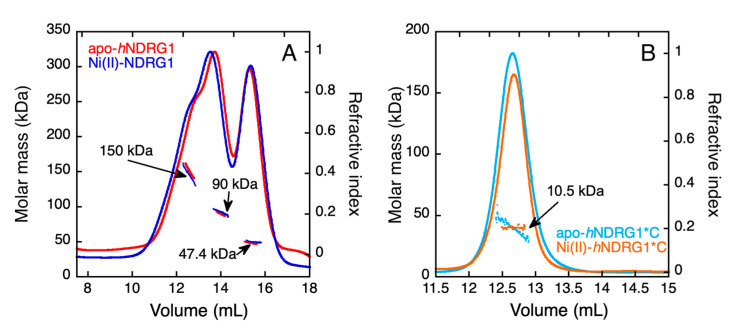
Molar mass and hydrodynamic radius determined by static and dynamic light scattering in line with a size-exclusion chromatography column. The chromatogram represents the trace of refractive index detector (lines) and the weight-averaged molar mass distribution, calculated on the eluting species, is represented as dots. The profile of *h*NDRG1 (**A**) and of *h*NDRG1*C (**B**) is reported in the absence and in the presence of four equivalents of Ni(II).

The hydrodynamic and oligomeric properties of *h*NDRG1 and *h*NDRG1*C in solution were determined using multiple-angle light scattering (MALS) and quasi-elastic light scattering (QELS) in line with a size-exclusion chromatography column (SEC). Elution of *h*NDRG1 occurred in three different peaks, corresponding to different oligomeric states of the protein in solution (Figure 10A). The calculated molar masses and hydrodynamic radii were MW = 150 kDa and R_h_ = 5.3 nm, respectively, for the first eluted species. The obtained MW value is intermediate between the molar mass of the tetramer (180 kDa) and that of the trimer (135 kDa). As this species is largely superimposed to the oligomeric form with lower molar mass, an underestimation of the calculated molecular weight is expected, as also supported by the profile shown by the dots, each representing the molar mass calculated for every slice under the peaks. This observation strongly suggests that the first eluted species is indeed the tetrameric form. The second eluting peak features MW = 90.5 kDa and R_h_ = 4 nm, corresponding to the values expected for the protein dimer. The last eluting peak is associated to MW = 47.4 kDa and R_h_ = 2.4 nm, corresponding to the expected values for the protein monomer (theoretical MW = 45 kDa). The oligomeric equilibrium observed in solution is not significantly altered in the presence of Ni(II) (Figure 10A). No significant change in the elution volume was observed when performing the SEC experiment in the presence of 10 mM DTT, excluding that the higher oligomeric states are formed by covalent disulfide bonds. 

Differently from the full-length protein, elution of *h*NDRG1*C from the SEC-MALS-QELS flow occurs as a unique peak with the characteristic of the monomer (MW = 10.5 kDa, R_h_ = 1.7 nm; theoretical MW = 8.6), both in the absence and in the presence of Ni(II) (Figure 10B), indicating that the full protein sequence is necessary to reach a multimeric form.

### 3.7. In-Cell Experiments

**Figure 11:**
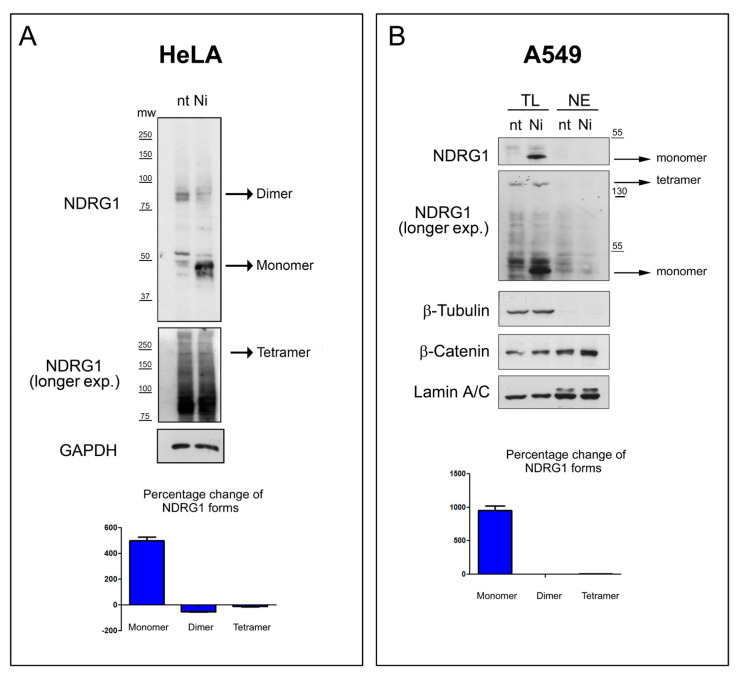
Nickel exposure promotes the expression of the monomeric form of *h*NDRG1. (**A**) HeLa cells were treated with 1 mM NiSO_4_ for 24 h or left untreated (nt) and lysed under nondenaturing conditions, and lysates were then resolved by an SDS-PAGE under nondenaturing conditions. Corresponding filters were incubated with an antibody against the N-terminal region of NDRG1. GAPDH was used as equal loading marker. Histogram indicates the fold variation of the indicated forms of NDRG1 after nickel exposure compared to the untreated counterparts. (**B**) Lung carcinoma-derived A549 cells were treated with 1 mM NiSO_4_ for two days (Ni) or left untreated (nt). Total lysates (TL) and nuclear extracts (NE) were analyzed in SDS-PAGE and the amount of NDRG1 was evaluated by Western blot analysis. β-tubulin and lamin A/C indicated the purity of nuclear extraction and equal loading. Histogram indicates the fold variation of the indicated forms of *h*NDRG1 after nickel exposure compared to the untreated counterparts. Western blots represent the most representative images of four repetitions of the same experiment.

In order to confirm the physiological relevance of *h*NDRG1 oligomeric equilibrium observed in solution, the expression profile and oligomeric state of endogenous *h*NDRG1 was assayed in two human cellular lines, Hela and A549—the latter derived from human lung carcinoma—in the absence and in the presence of NiSO_4_. At the end of the metal exposure, cells were lysed under nondenaturing conditions to preserve the stability of the *h*NDRG1 oligomers, and cellular lysates were resolved on SDS-PAGE under both denaturing and reducing conditions and nondenaturing conditions. For HeLa cells (Figure 11A), two bands at ca. 50 kDa and at ca.100 kDa, likely corresponding to the monomeric and the dimeric forms of the protein, were easily detectable. Of note, it is possible that all the faint bands around 50 kDa-band corresponded to different phosphorylation states of *h*NDRG1 [42] or of an N-terminal truncated form [38]. At longer exposure of the film, a faint band at ca. 200 kDa corresponding to the tetrameric form of *h*NDRG1 was also detectable. When HeLa cells were cultured with Ni(II), an increase in the level of expression of the monomeric form of *h*NDRG1 and a corresponding decrease in the band corresponding to the dimer are visible, suggesting that the presence of the metal ion shifts the equilibrium toward the low-molecular-weight states.

Differently from HeLa cells, no dimeric form of *h*NDRG1 was detected in A549 cells under these conditions, while the monomeric and the tetrameric forms were well-visible both in the absence and in the presence of Ni(II) (Figure 11B). Similarly to what was observed for the HeLa cells, addition of Ni(II) caused a marked expression of the monomeric specie. No form of *h*NDRG1 was found in the nucleus, both in the absence or in the presence of Ni(II), indicating that in this cellular line and under the experimental conditions the localization of *h*NDRG1 is cytoplasmatic. As previously reported, Ni(II) exposure increased the expression of b-catenin [91] (Figure 11B).

## 4. Discussion

Nickel is an essential element for unicellular organisms and plants, being responsible for the catalytic activity of several enzymes, many involved in bacterial pathogenesis [92], and being tightly regulated intracellularly [93]. For humans, nickel is considered a dangerous metal ion, responsible of several pathologies such as immunotoxicity and cancer [94]. The carcinogenic effect of nickel for the respiratory tissues has been observed for more than thirty years, but the molecular mechanisms that cause nickel-driven carcinogenesis are still unclear [95]. Ni(II)-induced cellular damage occurs mainly through epigenetic mechanisms [5]. One of the possible pathways is the ability of Ni(II) to substitute cognate Fe(II) ions in metal-binding enzymes responsible for a balanced epigenetic landscape and for the regulation of gene expression [96]. Understanding how Ni(II) binds its intracellular targets is thus a necessary step to unravel the molecular basis of its carcinogen effects and to develop antitumoral drugs for detoxifying it.

One of the promising intracellular targets for Ni(II) is *h*NDRG1 [12,28], a protein that is induced by Ni(II) through the hypoxia response pathway, showing an oncogenic effect in lung carcinomas and responding to iron-chelation therapy [15]. *h*NDRG1 contains a unique C-terminal region (*h*NDRG1*C) of 83 residues reported to be very flexible and able to bind Ni(II) [39]. The intrinsically disordered behavior of this protein portion is reflected in its primary structure, which shows high content of charged and hydrophilic residues and low abundance of hydrophobic amino acids. It is known that IDRs, identified in all living organisms, play important roles in the regulation of cellular metabolisms and gene expression, usually present very large interactomes and are linked to the progress of several diseases such as cancer [97,98,99]. Head and neck cancer cells transfected with the *h*NDRG1 gene truncated in the sequence coding for the C-terminal 338–394 residues showed remarkable lower migration and invasion abilities, as compared to the same cells transfected with the full-length *h*NDRG1 gene, indicating that the C-terminal IDR plays a crucial role for facilitating cell motility, a hallmark for cancer metastasis and progression [46]. In addition, deletion of this sequence abolished *h*NDRG1 nuclear translocation, which was reported to promote motility [46], also supporting the role of *h*NDRG1*C for promoting the carcinogenic process. In the present work, we characterized the flexible behavior of *h*NDRG1*C and studied its Ni(II) binding activity.

The protocol for the overexpression and purification to homogeneity of the wild-type *h*NDRG1*C, initially fused to a N-terminal ZZ-tag and subsequently cleaved, is reported. The absence of stable secondary and tertiary structures was experimentally proven using CD and NMR spectroscopies. In particular, the far-UV CD spectra were quantitatively analyzed and are typical of a highly flexible polypeptide with almost 70% of the protein structure attributed to random coil conformation. The ^1^H ^15^N HSQC spectra of *h*NDRG1*C are characteristic of an intrinsically disordered protein, with low signal dispersion in the ^1^H dimension. This observation is maintained from pH 6.5 to 7.5 and confirms that this region is dominated by random coil conformations, lacking a well-defined structure, as predicted by the in silico disorder prediction analysis. Assignment of the NMR signals, initially performed in the ^1^H,^15^N-HSQC spectrum at pH 6.5 then translated to the more physiological pH 7.5, and analysis of the secondary structure propensity from the chemical shift analysis, confirmed the prevalence of random coil structures, with a small helical propensity in the N-terminal region.

In solution, *h*NDRG1*C behaves as a monomer. Differently, SEC-MALS data show that the full-length *h*NDRG1 exists in solution in equilibrium between three oligomeric forms: tetramer, dimer and monomer. These forms were also identified for the natively expressed protein in lung adenocarcinoma and in HeLa cells, implying that the oligomeric states observed in solutions are not an artifact of the experimental conditions, such as the high protein concentration. The homologous protein *h*NDRG3 was reported to form dimers in solution [34], while *h*NDRG2 was observed as a monomer [33]. A similar SEC-MALS analysis on the full-length and truncated variants of *h*NDRG1 showed that these proteins eluted mostly in a single peak corresponding to the monomeric form, while a minor peak, attributed to a dimer, was observed for two truncated variants [35]. No tetrameric form was observed under these conditions, likely because the protein amount injected was ca. 20 times lower as compared to the present work (100 µg in [35] vs. 1.9 mg in this work), which implies that a significantly lower protein concentration was attained in the SEC column. This observation is consistent with a concentration-dependent oligomeric equilibrium.

In the hypothesis that Ni(II) ions exert a physiological or pathological cellular role through binding to *h*NDRG1, Ni(II)-binding capacity of this protein was previously studied using ITC, showing a single binding event with *K_D_* at ca. 100 µM at pH 7.0 [35]. A similar binding event was observed for a truncated variant lacking the C-terminal region, leading the authors of this past study to conclude that the Ni(II)-binding site of *h*NDRG1 was located in the globular α/β hydrolase-like domain and not in the C-terminal region [35]. This observation disagrees with previously reported experiments, which indicated that the three-fold-repeated decapeptide (3R-motif) contained in *h*NDRG1*C is able to bind Ni(II) in a square planar diamagnetic coordination using the His imidazole ring and three amides from the protein backbone [30,39]. Indeed, the data on the full-length protein could have been affected by the presence, in the recombinant *h*NDRG1, of a non-native His2 residue, deriving from the cloning procedure and the subsequent TEV protease cleavage of the N-terminal His-tag used for the purification [35]. This residue typically forms a Ni-hook that is known to have substantial Ni(II) binding affinity, forming a non-native metal-binding site that could shield or alter the physiological Ni(II)-binding site in *h*NDRG1, located in *h*NDRG1*C [100]. On the other hand, previous studies on the Ni(II)-binding activity on the 3R-motif did not consider the entire *h*NDRG1*C region; rather, they only analyzed the repeated sequence containing three histidine residues, corresponding to His335, His345 and His355 [30,39]. Notably, these studies reported the ability of the 3R-motif to bind three Ni(II) ions, with each decapeptide repeat being the minimum motif for Ni(II) binding, implying that there is not any chelation effect [30,39]. The results of these studies are affected by the absence, in the investigated peptides, of an additional histidine (His371) and a C-terminal Cys394, potential Ni(II) binding residues that are instead present in the primary structure of *h*NDRG1*C.

In the present study, the Ni(II)-binding capacity of the *entire h*NDRG1*C was confirmed and investigated using ITC and NMR. ITC data, obtained by averaging the thermodynamic parameters derived from four independent experiments, clearly show that *h*NDRG1 is able to bind 1–2 Ni(II) ions in a single binding event with mild affinity (*K_D_* ca. 70 µM). The difficulty in establishing the exact stoichiometry using ITC is most likely due to the absence, in the protein primary structure, of Trp residues and to the presence of very few aromatic residues (one Tyr and three Phe), which makes significant the relative error in estimating protein concentration by absorbance at 280 nm. The binding is enthalpically driven and shows negative entropy, suggesting that Ni(II) binding induces some conformational rearrangement. CD and NMR spectroscopies, however, did not reveal any major change in the protein backbone upon Ni(II) binding, nor any acquisition of structure of the disordered protein region, which was previously suggested [35]. The content of Ni(II) in lung tissue, measured as 20 ng/g and 8–330 ng/g of wet tissue in non-occupationally exposed subjects [101,102], corresponds to an approximative Ni(II) intracellular concentration ranging from 80 nM to 1.25 µM. Higher concentrations are possible for people inhaling nickel compounds from pollution, cigarette smoke or occupational exposure. Thus, the affinity measured for *h*NDGR1*C might be significant, especially under pathological conditions.

Ni(II) binding results in the disappearance of several signals in the diamagnetic region of the NMR spectra, assigned to the side-chain and backbone nuclei of the four histidine residues found in the protein sequence, as well as to residues immediately preceding and following them; moreover, the NMR signals of nuclei belonging to the side chains of one aspartate, three glutamates and one asparagine residue, as well as those of the terminal single cysteine, also disappear upon Ni(II) binding. This observation suggests that Ni(II) is bound to *h*NDRG1*C in an octahedral or square-pyramidal geometry, resulting in a paramagnetic metal site. This conclusion was confirmed by the observation of large hyperfine shifts in NMR spectra tailored for the detection of fast-relaxing proton signals. The large number of residues experiencing the paramagnetic effect of the bound metal ion suggests the coexistence of different metal-bound conformation in solution separated by a relatively flat energy landscape and possibly undergoing intramolecule metal transfer between different binding sites. This situation has been observed as typical for IDPs or IDRs, often showing a low affinity and highly dynamic binding sites for metal ions [103,104]. 

In addition to the binding of Ni(II) [30,39], a direct interaction of the 3R-peptide has been observed with Cu(II) [40], Zn(II) [105], Co(II) and Mn(II) [106]. In all cases, the binding center of the peptide fragment is associated with histidine and glutamate residues. Even though these studies clearly indicate the ability of the C-terminal region of *h*NDRG1 to interact with diverse divalent metal ions, they cannot be used to compare the results with the Ni(II)-binding affinity found here for *h*NDRG1*C, due to the difference in primary structure between the 3R-motif and the *h*NDRG1*C sequence, as described above. In addition, while Ni(II) and Co(II) induce the expression of *h*NDRG1 [107], as well as Fe(II) chelation [108], no in vivo effect has been reported for Cu(II), Mn(II) and Zn(II). Interestingly, a previous study showed that Ni(II) and Co(II) were able to affect the stability of the isolated full-length *h*NDRG1, while Fe(II), Fe(III) and Mg(II) did not show any effect and the isolated protein precipitated in the presence of Zn(II) [35].

It is known that *h*NDRG1*C is phosphorylated in vivo on serine or threonine residues, this post-translational modification strongly determining its ability to promote nuclear localization of *h*NDRG1 and cell migration [46]. It is likely that this important change in protein functionality reflects a significant modification of protein folding and/or interactions such as Ni(II)-binding affinity that can be affected by the presence of covalently bound phosphate groups. This observation can give reason of the relatively low Ni(II)-binding affinity reported for nonphosphorylated *h*NDRG1*C and *h*NDRG1 [35]. The study of the conformational changes associated to *h*NDRG1*C phosphorylation is currently under development in our laboratory.

## Figures and Tables

**Table 1:** Chemical shift (ppm) signal assignment of the histidine residues in hNDRG1*C at pH 6.5 and pH 7.5.

	His335		His345		His355		His371	
	*pH 6.5*	*pH 7.5*	*pH 6.5*	*pH 7.5*	*pH 6.5*	*pH 7.5*	*pH 6.5*	*pH 7.5*
**(H)N**	121.16	121.89	121.29	121.89	121.29	121.78	118.11	118.43
**C**	175.24	175.69	175.24	175.69	175.20	175.60	174.54	175.14
**CA**	56.13	56.67	56.13	56.67	56.26	56.65	55.56	56.20
**CB**	30.14	31.03	30.14	31.03	30.07	30.95	29.42	30.40
**HD2**	7.13	6.99	7.13	6.99	7.13	6.99	7.16	6.99
**CD2**	119.97	119.86	119.97	119.86	119.97	119.86	120.0	119.86
**HE1**	8.05	7.75	8.05	7.75	8.08	7.75	8.23	7.83
**CE1**	137.97	138.66	137.97	138.66	137.90	138.66	137.5	138.51

## Supplementary Materials

The following supporting information can be downloaded at: https://www.mdpi.com/article/10.3390/biom12091272/s1.
