# Supplementary material for: The Ni(II)-Binding Activity of the Intrinsically Disordered Region of Human NDRG1, a Protein Involved in Cancer Development
Source: Biomolecules. 2022 Sep 9;12(9):1272. doi: 10.3390/biom12091272 (PMC9496542; doi:10.3390/biom12091272)
Supplement: Supplementary file 1 [file biomolecules-12-01272-s001.zip › biomolecules-1865674-supplementary.pdf]

The Ni(II) binding activity of the intrinsically disordered region of human NDRG1, a protein involved in cancer development

Ylenia Beniamino[a], Vittoria Cenni[b], Mario Piccioli[c], Stefano Ciurli[a*], Barbara Zambelli[a*]

[a] *Department of Pharmacy and Biotechnology, Laboratory of Bioinorganic Chemistry, University of Bologna: Viale Giuseppe Fanin 40, 40127Bologna, Italy,*

[b] *CNR Institute of Molecular Genetics "Luigi-Luca Cavalli-Sforza" Unit of Bologna, Via di Barbiano 1/10, 40136 Bologna, Italy*

[c] *Department of Chemistry, Center for Magnetic Resonance, University of Florence, 50121 Florence, Italy;*

[*] Corresponding authors: stefano.ciurli@unibo.it; barbara.zambelli@unibo.it

SUPPLEMENTARY MATERIALS

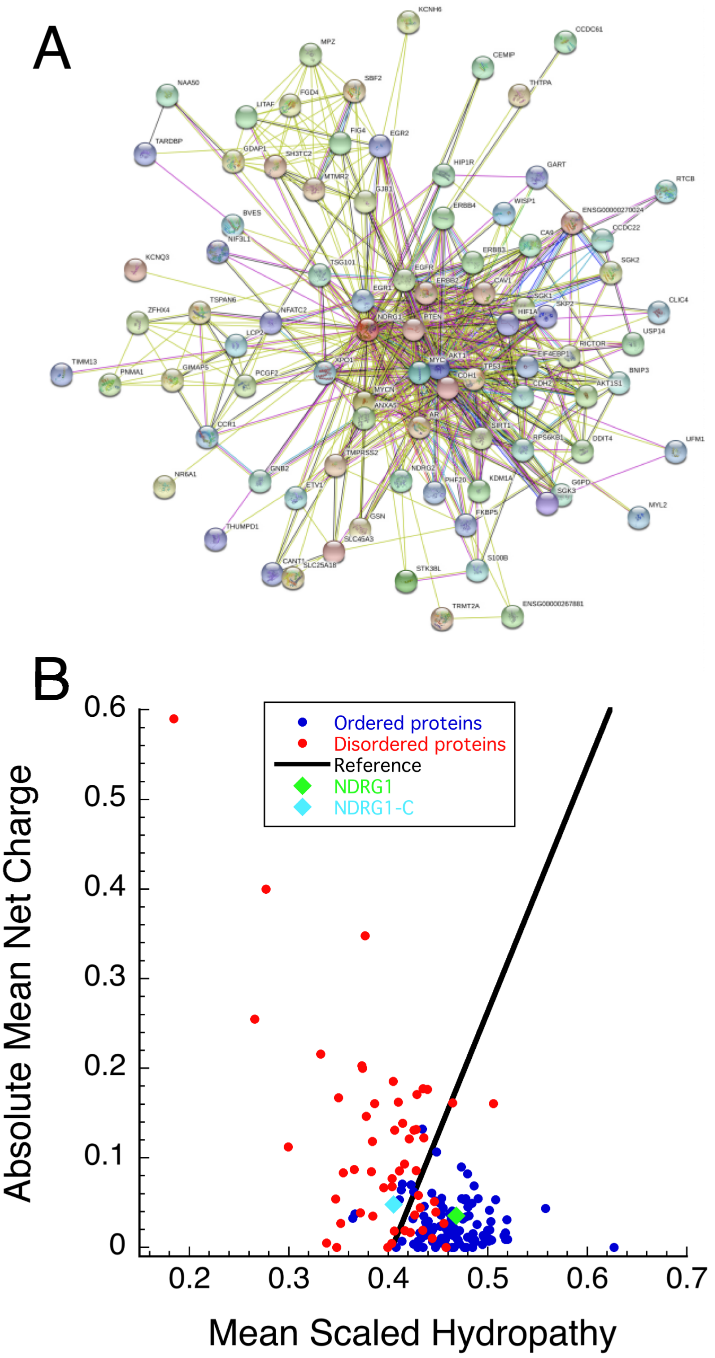

Figure S1. A) Visualization of the interactivity of *h*NDRG1 by the STRING computational platform. **B**) Relation between mean net charge and hydropathy for *h*NDRG1 and *h*NDRG1*C represented in the CH-plot, predicting the ordered or disordered nature of both proteins.

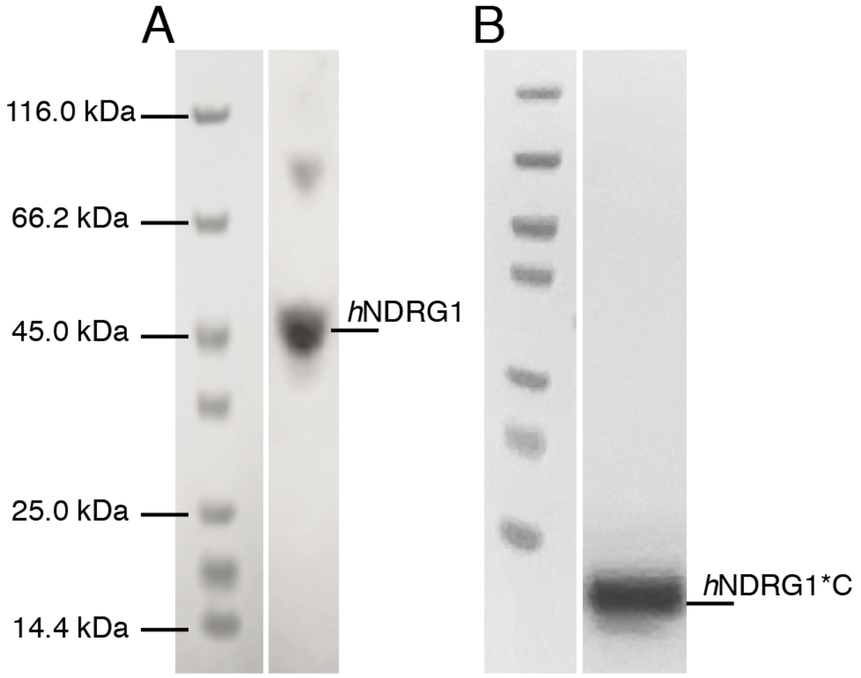

Figure S2. SDS-Page of the purified *h*NDRG1 (**A**) and *h*NDRG1*C (**B**) after the last size-exclusion chromatographic step. The molecular weight marker with the corresponding MW is represented on the left side of each line.

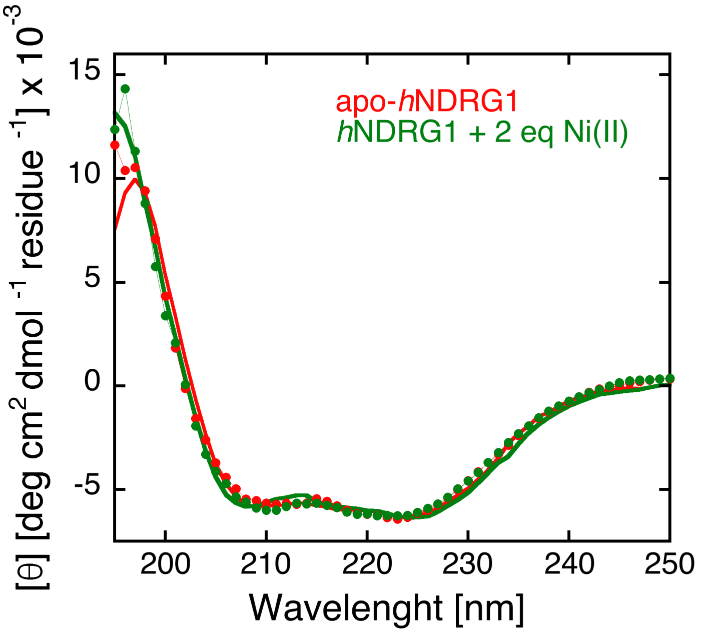

Figure S3. Far-UV CD spectra of *h*NDRG1 in the absence (red) and in the presence (green) of Ni(II). The fit of the data performed using BestSel is indicated as a line.

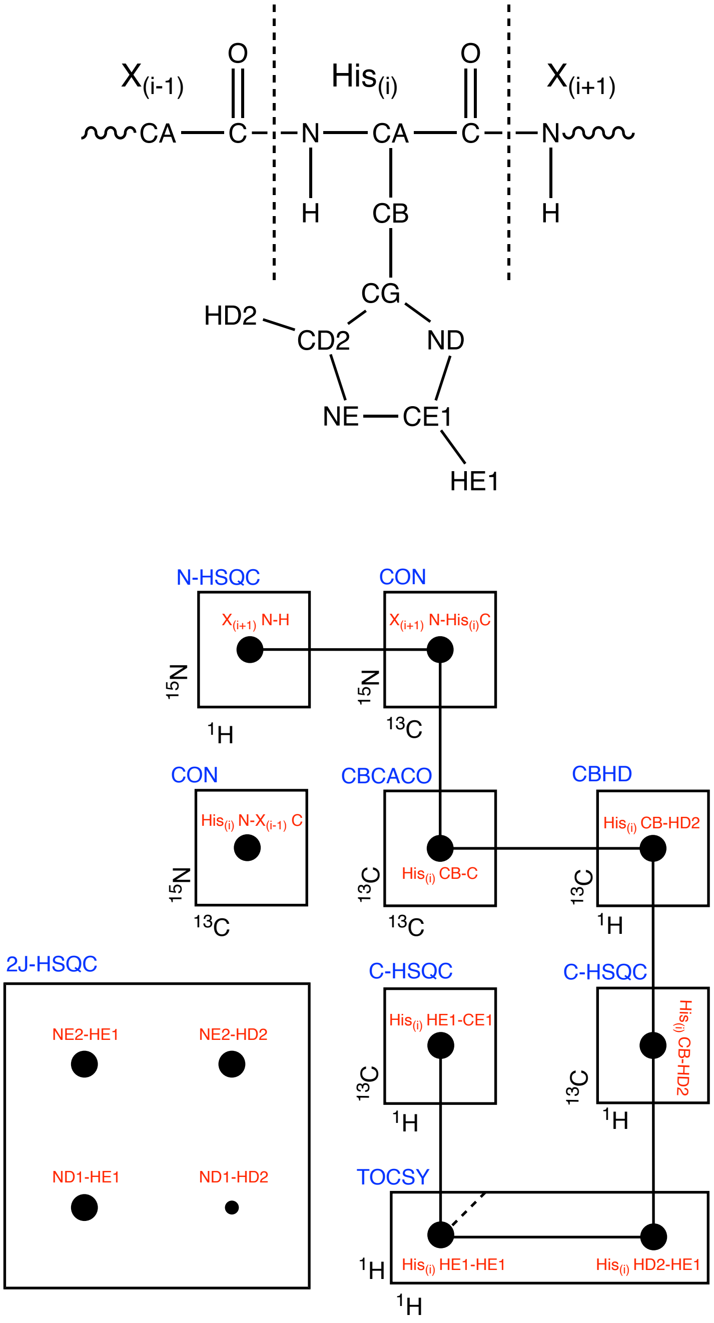

Figure S4. Scheme used for the full assignment of the NMR signals of the His residues in *h*NDRG1*C. The unambiguous assignment of each His(i) residue starts from the signal of the X(i+1) amide N nucleus in the ^{1}H,^{15}N HSQC spectrum, which allows the identification of the carbonyl C signal of His(i) in the CON spectrum. In turn, this grants the identification of the CB signal in the CBCACO spectrum; knowledge of the position of His(i) CB then permits the identification of the side chain imidazole HD2 signal in the CBHD spectrum, and consequently the recognition of the signal for CD2 using the ^{13}C HSQC focused on the aromatic region, the signal for HE1 using the ^{1}H,^{1}H TOCSY, and finally signal of CE1 using again the aromatic ^{13}C HSQC spectrum. The signal of the amide ^{15}N nucleus of His(i) could then be assigned by observing its correlation with the carbonyl C of the X(i-1) residue in the CON spectrum. Finally, 2J ^{1}H,^{15}N-HMQC spectra allowed the assignment of the ^{15}N signals of all NE2 and ND1 nuclei of histidines imidazoles.

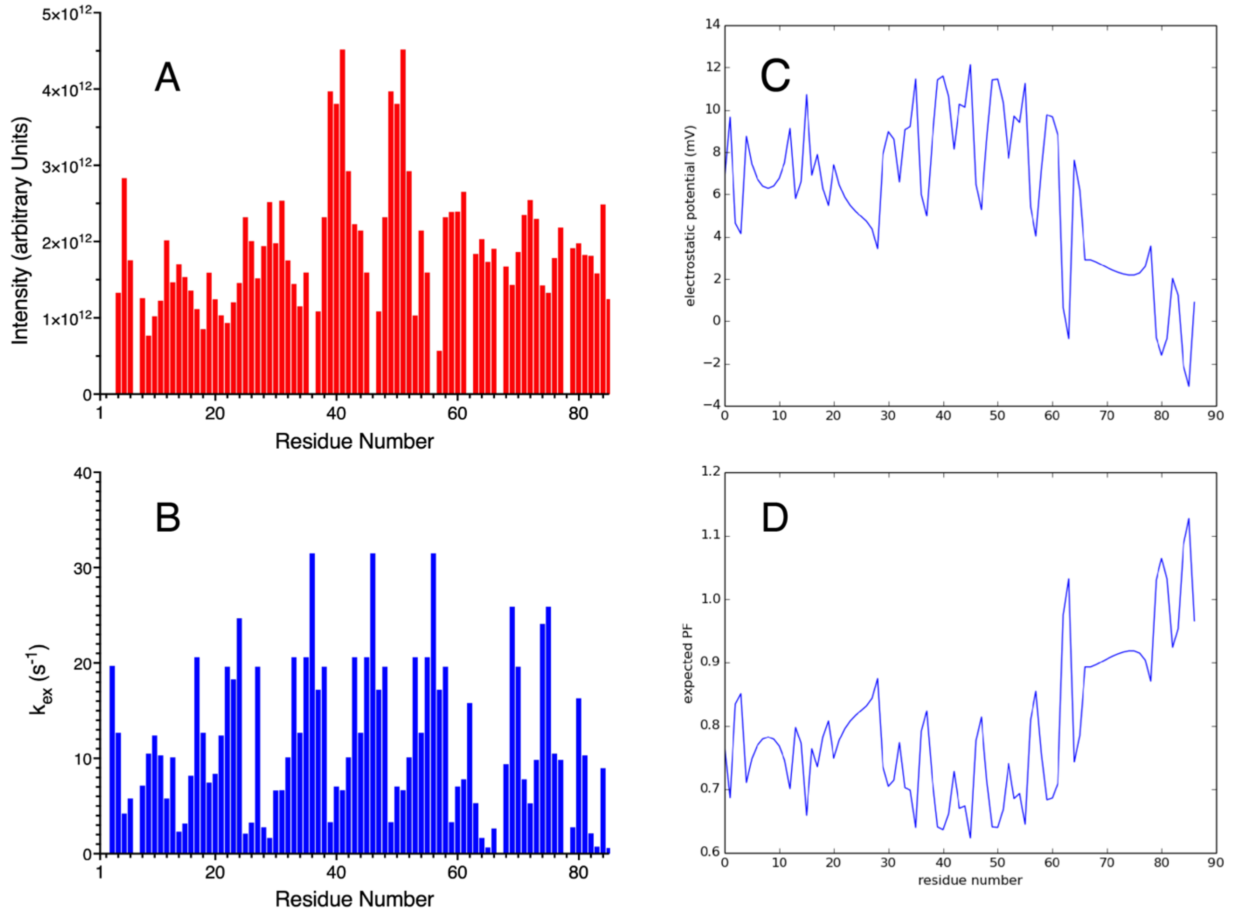

Figure S5. (**A**) *h*NDRG1*C amide NH signal intensities at pH 6.5 as resulting from the $^{1}H,^{15}N$ HSQC spectrum; (**B**) intrinsic exchange rate for that residue predicted using SPHERE (https://protocol.fccc.edu/research/labs/roder/sphere/sphere.html); (**C**) electrostatic potential and (D) protection factor calculated using the recently proposed approach by Mulder et al. [1]

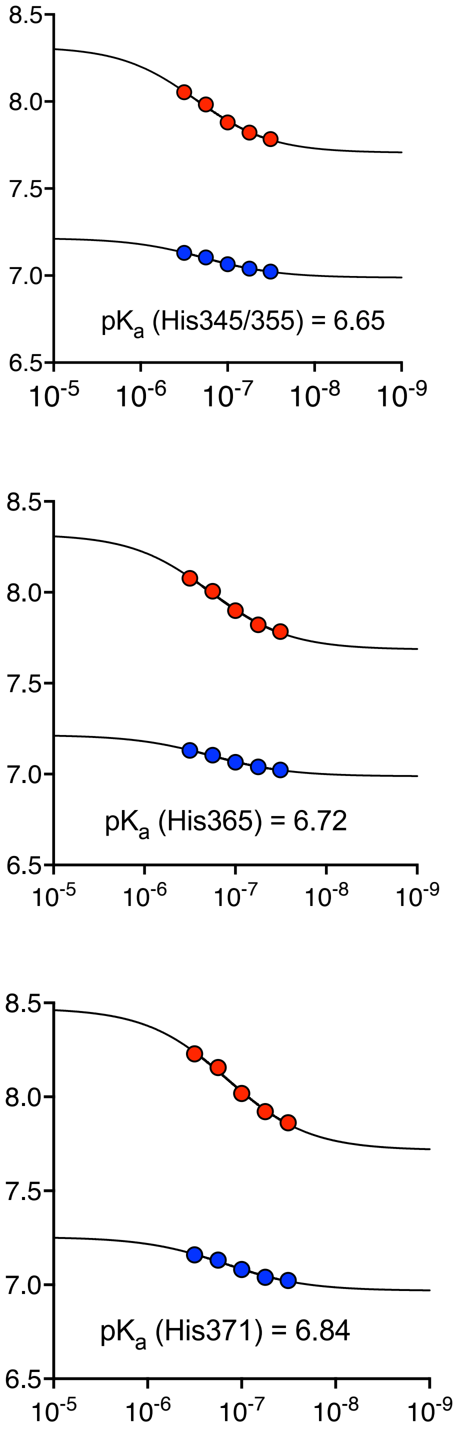

Figure S6. pH dependence of the chemical shifts of HE1 (red dots) and HD2 (blue dots) protons for the four histidine residues in *h*NDRG1*C, and the calculated values of the individual pKa obtained from simultaneous non-linear fits of to the following one-ionization equation (Eq. 1)

$$\delta_{obs} = \frac{[H^+] \cdot \delta_{HisH} + K \cdot \delta_{His}}{[H^+] + K} \tag{1}$$

where δ_{obs} is the observed experimental chemical shift, δ_{HisH} and δ_{His} are the chemical shifts of the protonated and neutral forms of the histidine imidazole, and K is the dissociation constant for the ionization equilibrium.

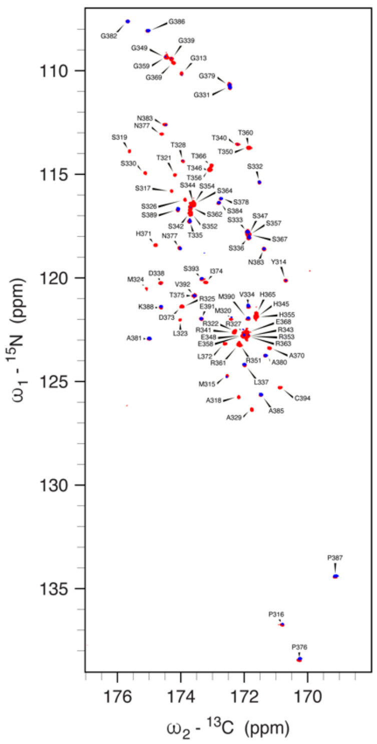

Figure S7. 700 MHz (16.4 T) 2D CON spectrum obtained by ^{13}C direct detection, acquired at 298 K on samples of ^{13}C,^{15}N labeled *h*NDRG1-C, at pH 7.5 in the absence (red) and presence (blue) of 3 eq. Ni(II). The resonances are labeled according to the ^{15}N frequency.

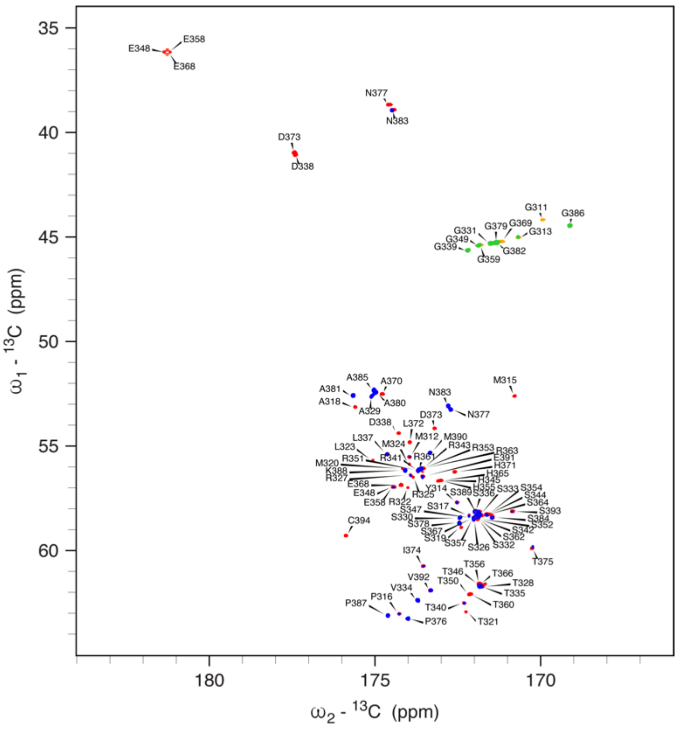

Figure S8. 700 MHz (16.4 T) 2D CACO spectrum obtained by ^{13}C direct detection, acquired at 298 K on samples of ^{13}C,^{15}N labeled *h*NDRG1-C, at pH 7.5 in the absence (red positive, orange negative) and presence (blue positive, green negative) of 3 eq. Ni(II). The resonances are labeled according to the ^{13}C frequencies of the CO and CA nuclei of each amino acid, except for the signals for the side chains of Asp and Glu residues, for which the CB-CG and CG-CD are explicitly indicated.

Table S1. NMR experiments carried out on ^{13}C,^{15}N labelled NDRG1-C in 95% H_2O / 5% D_2O at 298 K and pH 6.5 for backbone resonance assignment. SW: spectral width in each dimension; TD: number of complex points in each dimension; NUS: amount of sparse sampling (if not indicated conventional sampling was used); CT = constant time

**NMR experiment**			
^{1}H-^{15}N HSQC	^{1}H	^{15}N	
SW (Hz)	10416.667	2923.977	
maximal evolution time (ms)	98.3	43.8	
TD	2048	256	
^{1}H-^{1}H TOCSY	^{1}H	^{1}H	
SW (Hz)	11904.762	11904.762	
maximal evolution time (ms)	6.5	2.7	
TD	1536	640	
^{1}H-^{13}C HSQC	^{1}H	^{13}C	
SW (Hz)	10416.667	50000.000	
maximal evolution time (ms)	98.3	5.12	
TD	1024	512	
^{1}H-^{13}C HSQC CT	^{1}H	^{13}C	
SW (Hz)	19230.770	22727.273	
maximal evolution time (ms)	53.2	11.3	
TD	2048	512	
^{1}H-^{13}C HSQC aromatic	^{1}H	^{13}C	
SW (Hz)	11904.762	9090.909	
maximal evolution time (ms)	6.5	14.1	
TD	1536	256	
^{1}H-^{13}C HSQC CT aromatic	^{1}H	^{13}C	
SW (Hz)	11904.762	9090.909	
maximal evolution time (ms)	6.5	14.1	
TD	1536	256	
hbCBcgcdHD (CBHD)	^{1}H	^{13}C	
SW (Hz)	11904.762	6250.000	
maximal evolution time (ms)	6.5	8.5	
TD	1536	106	
HNCO	^{1}H	^{15}N	^{13}C
SW (Hz)	10416.667	2923.977	2702.703
maximal evolution time (ms)	196.6	87.6	47.4
TD	4096	512	256
NUS (%)	4		
HN(CA)CO	^{1}H	^{15}N	^{13}C
SW (Hz)	10416.667	2923.977	2702.703
maximal evolution time (ms)	196.6	87.6	47.4
TD	4096	512	256
NUS (%)	4		
HNCA	^{1}H	^{15}N	^{13}C
SW (Hz)	10416.667	2923.977	8333.333
maximal evolution time (ms)	98.3	23.9	11.3
TD	2048	128	192
NUS (%)	9.8		
HNCACB	^{1}H	^{15}N	^{13}C
SW (Hz)	10416.667	2923.977	22727.273
maximal evolution time (ms)	98.3	23.9	11.3
TD	2048	128	512
NUS (%)	4.9		
HN(CO)CACB	^{1}H	^{15}N	^{13}C
SW (Hz)	10416.667	2923.977	8333.333
maximal evolution time (ms)	98.3	23.9	11.3
TD	2048	128	512
NUS (%)	5		

CBCA(CO)NH	^{1}H	^{15}N	^{13}C
SW (Hz)	10416.667	2923.977	22727.273
maximal evolution time (ms)	98.3	23.9	6.6
TD	2048	128	300
NUS (%)	10		
HBHANH	^{1}H	^{15}N	^{1}H
SW (Hz)	10416.667	2923.977	6666.667
maximal evolution time (ms)	98.3	23.9	38.4
TD	2048	140	512
NUS (%)	4		
HBHA(CO)NH	^{1}H	^{15}N	^{1}H
SW (Hz)	10416.667	2923.977	6666.667
maximal evolution time (ms)	98.3	23.9	38.4
TD	2048	140	512
NUS (%)	4		
C(CO)NH	^{1}H	^{15}N	^{13}C
SW (Hz)	10416.667	2923.977	22727.273
maximal evolution time (ms)	98.3	23.9	11.3
TD	2048	128	512
NUS (%)	2		
(H)CCH-TOCSY	^{1}H	^{13}C	^{13}C
SW (Hz)	10416.667	22727.273	22727.273
maximal evolution time (ms)	98.3	28.2	11.3
TD	2048	128	512
NUS (%)	4		
HC(C)H-TOCSY	^{1}H	^{13}C	^{1}H
SW (Hz)	10416.667	22727.273	22727.273
maximal evolution time (ms)	98.3	28.2	5882.353
TD	1024	128	1024
NUS (%)	5		
hCON	^{13}C	^{15}N	
SW (Hz)	5555.556	2840.909	
maximal evolution time (ms)	92.2	90.1	
TD	1024	512	
hCACO	^{13}C	^{13}C	
SW (Hz)	5555.556	6369.427	
maximal evolution time (ms)	92.2	27.5	
TD	1024	350	
hCBCACO	^{13}C	^{13}C	
SW (Hz)	5555.556	11363.636	
maximal evolution time (ms)	98.3	22.5	
TD	1024	512	
2J HMQC	^{1}H	^{15}N	
SW (Hz)	11398.177	7692.308	
maximal evolution time (ms)	52.64	16.64	
TD	1200	256	

References

1. Dass, R.; Corliano, E.; Mulder, F. A. A., The contribution of electrostatics to hydrogen exchange in the unfolded protein state. *Biophys J* **2021,** *120* (18), 4107-4114.
